# Risk of drug-related aggression in pediatric populations: a pharmacovigilance analysis using the FAERS database

**DOI:** 10.3389/fped.2026.1803086

**Published:** 2026-06-05

**Authors:** Jing Chen, Lilan Zhao

**Affiliations:** 1 Department of Pharmacy, Fujian Children’s Hospital (Fujian Branch of Shanghai Children’s Medical Center), Fuzhou, China; 2 Department of Thoracic Surgery, Shengli Clinical Medical College of Fujian Medical University, Fujian Provincial Hospital, Fuzhou University Affiliated Provincial Hospital, Fuzhou, China

**Keywords:** disproportionality analysis, drug-related aggression, FAERS, pediatric populations, pharmacovigilance

## Abstract

**Background:**

Aggression in pediatric populations poses significant risks to mental health and development, and drug-associated aggression may either exacerbate pre-existing behavioral problems or arise *de novo* following medication exposure. Existing studies, primarily based on randomized controlled trials (RCTs) and cohort designs, often underestimate real-world incidence. This study used the FDA Adverse Event Reporting System (FAERS) to systematically identify drugs associated with pediatric aggression, examining age/sex-specific differences and time-to-onset (TTO) patterns.

**Methods:**

We conducted a retrospective disproportionality analysis of FAERS data (2004 Q1–2025 Q2) for patients <18 years. Aggression was defined using Standardized MedDRA Queries (SMQ code: 10001488). Signals were detected using four consensus algorithms (ROR, PRR, MGPS, and BCPNN). Drugs were categorized by Anatomical Therapeutic Chemical (ATC) classification. Subgroup analyses and multivariable logistic regression were used to calculate adjusted reporting odds ratios (aRORs), and TTO was evaluated to characterize temporal patterns.

**Results:**

Among 7,959 reports (67.75% male; median age: 9 years), 31 drugs exhibited positive signals, primarily nervous system (45.16%) and respiratory (32.26%) agents. Signals for tezacaftor, elexacaftor, macrogol, desloratadine, and ebastine were identified despite absent FDA label warnings. The strongest signals were for ebastine (ROR: 23.40) and perampanel (17.41), while montelukast had the highest case volume (*N* = 1,392). Most drugs showed the highest aRORs in early childhood, except levetiracetam, which peaked in adolescence. Females generally showed higher risks across most agents. Median TTO ranged from rapid (anti-infectives: 2–3 days) to delayed (montelukast: 133 days; macrogol: 129 days).

**Conclusion:**

This study identifies robust signals linking multiple drugs to pediatric aggression, with elevated risks observed particularly in younger children and females. These findings underscore the need for heightened clinical vigilance, consideration of demographic-specific risks, and updated regulatory labeling to improve pediatric drug safety.

## Background

Aggression is a prevalent behavioral disorder in children and adolescents that poses substantial challenges to mental health, social functioning, and long-term development. It is associated with elevated risks of academic difficulties, peer rejection, and psychiatric comorbidities, including conduct disorder and antisocial personality traits, particularly in socioeconomically vulnerable populations (1, 2). Drug-associated aggression represents a distinct subtype of this condition, manifesting as an adverse drug reaction that may exacerbate pre-existing issues or arise *de novo* (3). Unlike aggression attributable to neurodevelopmental, environmental, or genetic factors, medication-induced aggression often has subtle presentation and delayed onset, which can lead to underdiagnosis and prolonged exposure to the causative agent. Commonly implicated medications include psychotropic drugs, such as selective serotonin reuptake inhibitors (*e.g.*, fluoxetine) and stimulants used for attention-deficit/hyperactivity disorder (*e.g.*, methylphenidate) (4–7).

Although clinically important, comprehensive population-level investigations of medication-associated aggression in minors remain scarce. Most existing evidence derives from randomized controlled trials or observational cohorts, which frequently underestimate real-world rates due to restrictive enrollment, limited follow-up duration, and underrepresentation of subgroups such as preschool children or those with comorbidities (8). Additionally, many drugs with potential aggressogenic effects lack pediatric-specific behavioral warnings, as premarketing studies rarely prioritize neuropsychiatric endpoints in youth (9). This evidence gap impedes proactive risk management and personalized treatment approaches, underscoring the value of robust pharmacovigilance methods in real-world settings.

The U.S. Food and Drug Administration Adverse Event Reporting System (FAERS) is a critical tool for post-marketing surveillance, containing over 20 million spontaneous reports submitted by healthcare professionals, patients, and manufacturers (10). As a publicly available resource, FAERS enables detection of rare or delayed adverse reactions through disproportionality analysis, which compares the observed reporting frequency of a drug-event pair against database expectations (11). Its widespread use has facilitated global identification of previously underrecognized safety signals. The present study addresses this knowledge gap by performing a disproportionality analysis of FAERS data from the first quarter of 2004 to the second quarter of 2025. Using real-world evidence, we aimed to detect positive drug signals for aggression in minors, compare them with current labeling, and explore age- and sex-specific differences to inform clinical practice and regulatory decisions.

## Methods

### Data sources

Data were extracted from FAERS, a spontaneous reporting database with quarterly public updates. The study period spanned the first quarter of 2004 to the second quarter of 2025. Aggression-related reports were identified using the Medical Dictionary for Regulatory Activities (MedDRA) preferred term “aggression” as defined within the corresponding Standardized MedDRA Query (SMQ code: 10001488). The dataset included seven core tables: demographic (DEMO), drug (DRUG), therapy duration (THER), indications (INDI), reactions (REAC), reporter sources (RPSR), and outcomes (OUTC) (10).

### Data processing

**Figure 1:**
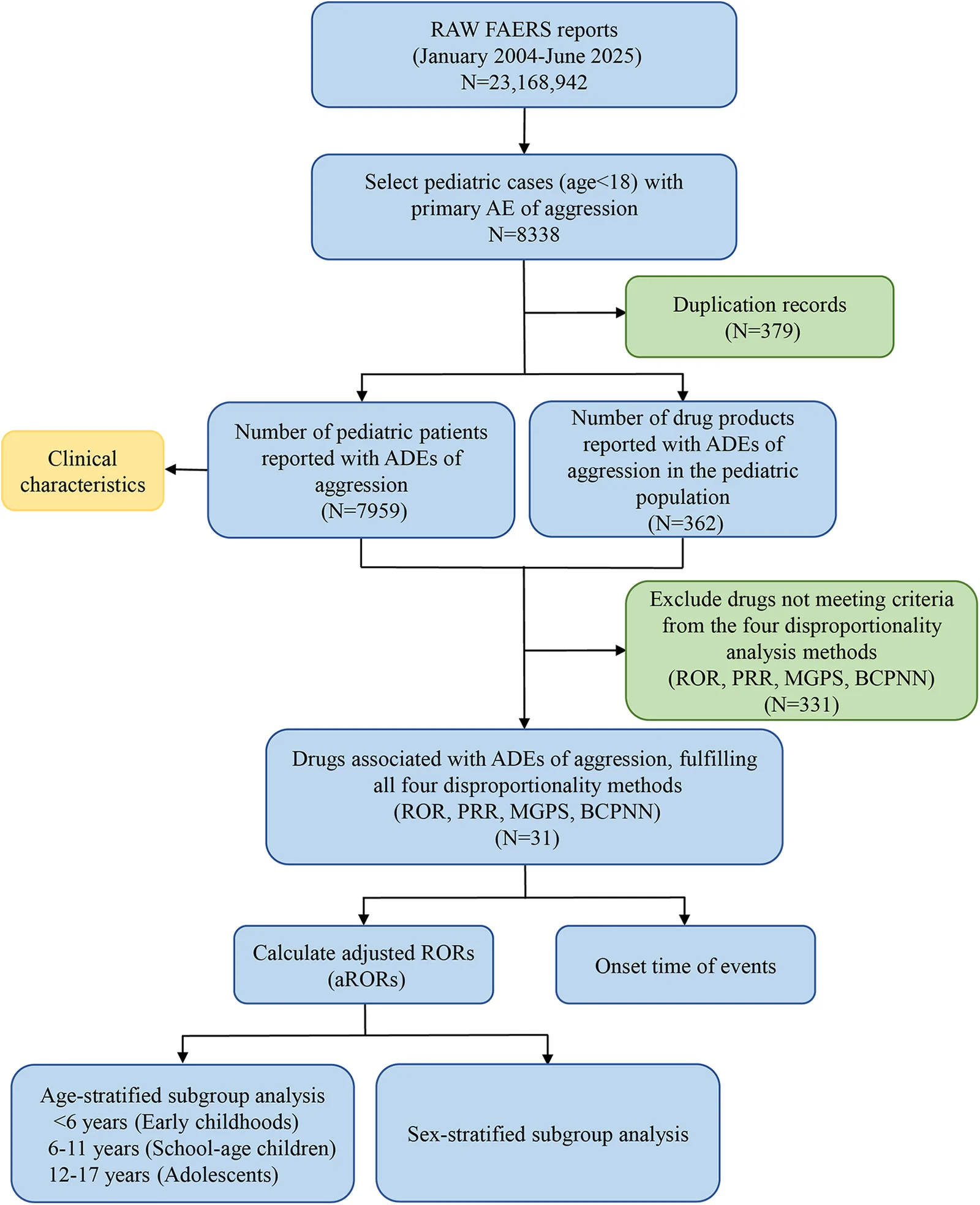
Workflow for data processing of pediatric drug-related aggression cases from the FDA adverse event reporting system (FAERS) database.

From 23,168,942 individual case safety reports (January 2004—June 2025), pediatric cases (age <18 years) with aggression listed as a primary adverse reaction (MedDRA version 28.0) were selected, yielding 8,338 initial records. After applying FDA-recommended deduplication (based on PRIMARYID, CASEID, and FDA_DT; retaining only the most recent report for each CASEID), 379 duplicates were removed. To maximize representativeness of the safety signals, reports with missing sex data were retained and categorized as “Unknown” rather than excluded. This resulted in a final dataset of 7,959 unique reports involving 362 primary suspect drugs. Drug names were standardized, and active substances were classified using the World Health Organization Anatomical Therapeutic Chemical (ATC) system. The overall data processing workflow is illustrated in Figure 1.

### Signal detection analysis

Positive signals were detected using four established disproportionality methods: reporting odds ratio (ROR), proportional reporting ratio (PRR), Bayesian confidence propagation neural network (BCPNN), and multi-item gamma Poisson shrinker (MGPS). For PRR, chi-square statistics and Fisher's exact test *p*-values were adjusted for false discovery rate (12). These algorithms are endorsed by major regulatory bodies, including the WHO-Uppsala Monitoring Centre and FDA (13, 14). Detailed formulas and threshold criteria are provided in Supplementary Table S1 and Supplementary Table S2. Specific thresholds were applied for each disproportionality method. A signal was considered positive if it met all of the following criteria: ROR025 > 1 and case count ≥ 3 for ROR; PRR ≥ 2 with *χ*² ≥ 4, case count ≥ 3, and PRR lower 95% CI > 1 for PRR; EBGM05 > 2 for MGPS; and IC025 > 0 for BCPNN. Statistical significance testing (*p* < 0.05) was not used as the primary criterion for signal detection, as it tends to be overly sensitive in large databases due to the influence of sample size; instead, confidence interval bounds served as the principal measure of disproportionality strength and robustness (15). Positive-signal drugs were then subjected to subgroup analyses. To evaluate whether aggression-related events were reflected in regulatory labeling, the “Warnings and Precautions” and “Adverse Reactions” sections of the current FDA prescribing information for each signal-positive drug were reviewed (DailyMed and FDA.gov, accessed March 2026) for mentions of aggression or related behavioral adverse events.

To address potential reporting biases due to geographic differences in FAERS, particularly for drugs with predominantly non-U.S. reports (*e.g.*, ebastine), we conducted a sensitivity analysis by restricting the background and control groups to non-U.S. reports only. This involved recalculating the disproportionality measures (ROR, PRR, MGPS, and BCPNN) using non-U.S. denominators. Signals were considered persistent if they remained positive across all four algorithms.

### Subgroup analyses

To account for potential confounders and derive adjusted reporting odds ratios (aRORs), binary multivariable logistic regression analyses were conducted, with aggression presence (yes/no) as the dependent variable and drug exposure (yes/no) as the primary independent variable. Models were adjusted for sex (male/female) or age group [early childhood (<6 years), school-age children (≥6 to <12 years), or adolescents (≥12 to <18 years)], as appropriate. Age groups followed the U.S. NICHD classifications; however, the “early childhood” category (<6 years) aggregates several distinct stages: neonates (0–27 days), infants (≥28 days to <1 year), toddlers (≥1 to <3 years), and the NICHD-defined early childhood (≥3 to <6 years). This merger was necessitated by the limited number of aggression reports in the youngest subgroups (particularly neonates and infants), ensuring sufficient statistical power and stable aROR estimation in multivariable logistic regression models. All comparisons were made relative to the entire FAERS database for patients aged <18 years not involving the specific drug of interest, rather than restricted to the drugs with positive signals for aggression included in the study (16). Logistic regression models were fitted separately for subgroup analyses to ensure covariate adjustment without interaction terms, unless specified. For age-stratified analyses, sex-adjusted aRORs and 95% confidence intervals (CIs) were calculated for each age group; similarly, for sex-stratified analyses, age-adjusted aRORs and 95% CIs were computed for males and females. Data preparation entailed constructing a case-level dataset, with each row representing a unique primary ID and including variables for aggression (binary: 1 = yes, 0 = no), drug exposure (binary: 1 = yes, 0 = no), sex (factor: male/female), and age group (factor: as defined above); missing values in key variables were addressed via listwise deletion, and datasets were verified for uniqueness, adequate sample size (total cases ≥100, aggression cases ≥20, and subgroup cases ≥5), and proper encoding prior to analysis. To assess differences in aRORs between sexes, sex-specific aRORs (adjusted for age) were compared using Z-statistics; likewise, to evaluate differences between age groups, age group-specific aRORs (adjusted for sex) were compared pairwise using Z-statistics, calculated as Z = (ln(aROR1)—ln(aROR2))/√(SE(ln(aROR1))^2^ + SE(ln(aROR2))^2^), where SE denotes the standard error. *P*-values were derived from the standard normal distribution, and to control for type I error due to multiple comparisons (*e.g.*, pairwise age group comparisons or comparisons across drugs), *p*-values were adjusted using the Holm method, with statistical significance defined as an adjusted *p*-value <0.05.

### Time-to-onset (TTO) analysis

TTO was computed as the interval between drug start date (START_DT) and event onset date (EVENT_DT), with records containing missing or implausible dates excluded. Median TTOs with interquartile ranges (IQRs) were reported, and cumulative incidence curves by ATC class were generated using Kaplan–Meier estimation.

### Statistical analysis

All analyses were performed in R (version 4.4.2). A two-sided *p*-value < 0.05 was considered statistically significant.

## Results

### Baseline characteristics

**Figure 2:**
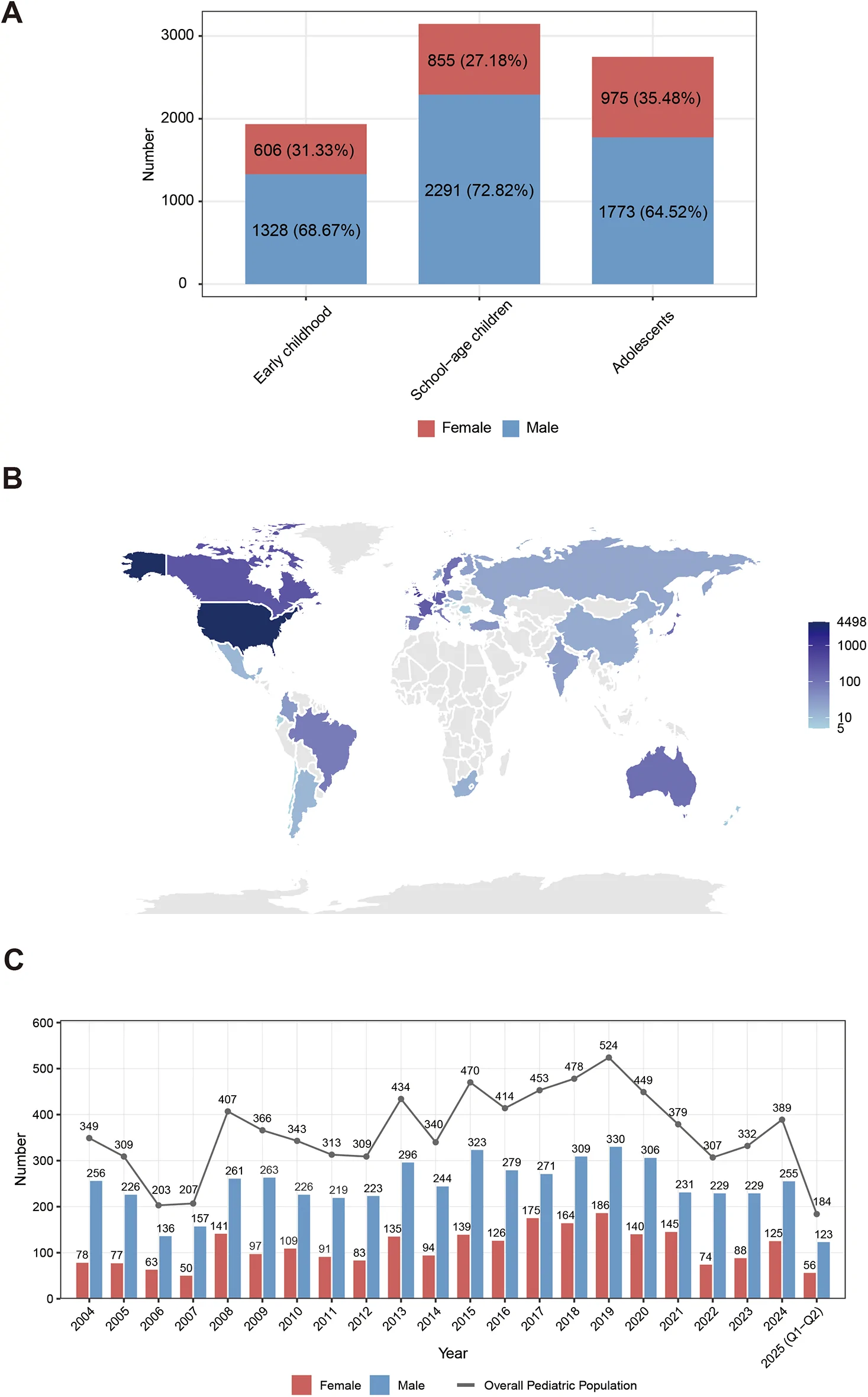
Baseline characteristics among pediatric patients with drug-related aggression in FAERS. **(A)** Age distribution by sex; **(B)** Geographic distribution; **(C)** Temporal trend of reporting.

The cohort comprised 7,959 unique reports of drug-associated aggression in patients aged <18 years (median 9 years, IQR 6–14), with no missing age data. Age distribution was 24.80% early childhood (<6 years), 40.14% school-age (≥6 to <12 years), and 35.05% adolescence (≥12 to <18 years). Regarding sex, males accounted for 67.75% of reports, females for 30.61%, and sex was unknown in 1.65% of cases; sex ratios were similar across the three age groups (Figure 2A). Consumers were the most common reporters (44.93%), followed by physicians (20.87%). The predominant outcome was “Other Serious” (45.80%), followed by hospitalization (17.68%). Reports originated primarily from the United States (56.51%), followed by the United Kingdom, Canada, France, and Germany (Figure 2B). The annual number of reports peaked in 2019, declined during 2020–2022, and subsequently increased from 2023 onward (Figure 2C). Detailed baseline characteristics are summarized in Table 1.

**Table 1:** Baseline characteristics of pediatric patients with aggression reported in the FAERS database.

Characteristics	Total (*N* = 7,959), *n* (%)
Age	
Median (IQR)	9.00 (6.00, 14.00)
Early childhood (<6 years)	1,974 (24.80%)
School-aged children (≥6 to <12 years)	3,195 (40.14%)
Adolescents (≥12 to <18 years)	2,790 (35.05%)
Gender	
Female	2,436 (30.61%)
Male	5,392 (67.75%)
Unknown	131 (1.65%)
Reporter's type	
Consumer	3,576 (44.93%)
Physician	1,661 (20.87%)
Pharmacist	330 (4.15%)
Lawyer	63 (0.79%)
Other healthcare professional	1,459 (18.33%)
Unknown	870 (10.93%)
Outcomes	
Other serious (important medical event)	3,645 (45.80%)
Hospitalization—initial or prolonged	1,407 (17.68%)
Disability	386 (4.85%)
Life—threatening	346 (4.35%)
Death	108 (1.36%)
Required intervention to prevent permanent impairment/damage	52 (0.65%)
Congenital anomaly	14 (0.18%)
Unknown	2,001 (25.14%)
Country (Top 5)	
United States	4,498 (56.51%)
United Kingdom	891 (11.19%)
Canada	296 (3.72%)
France	248 (3.12%)
Germany	205 (2.58%)

### Positive signal values of drugs

**Figure 3:**
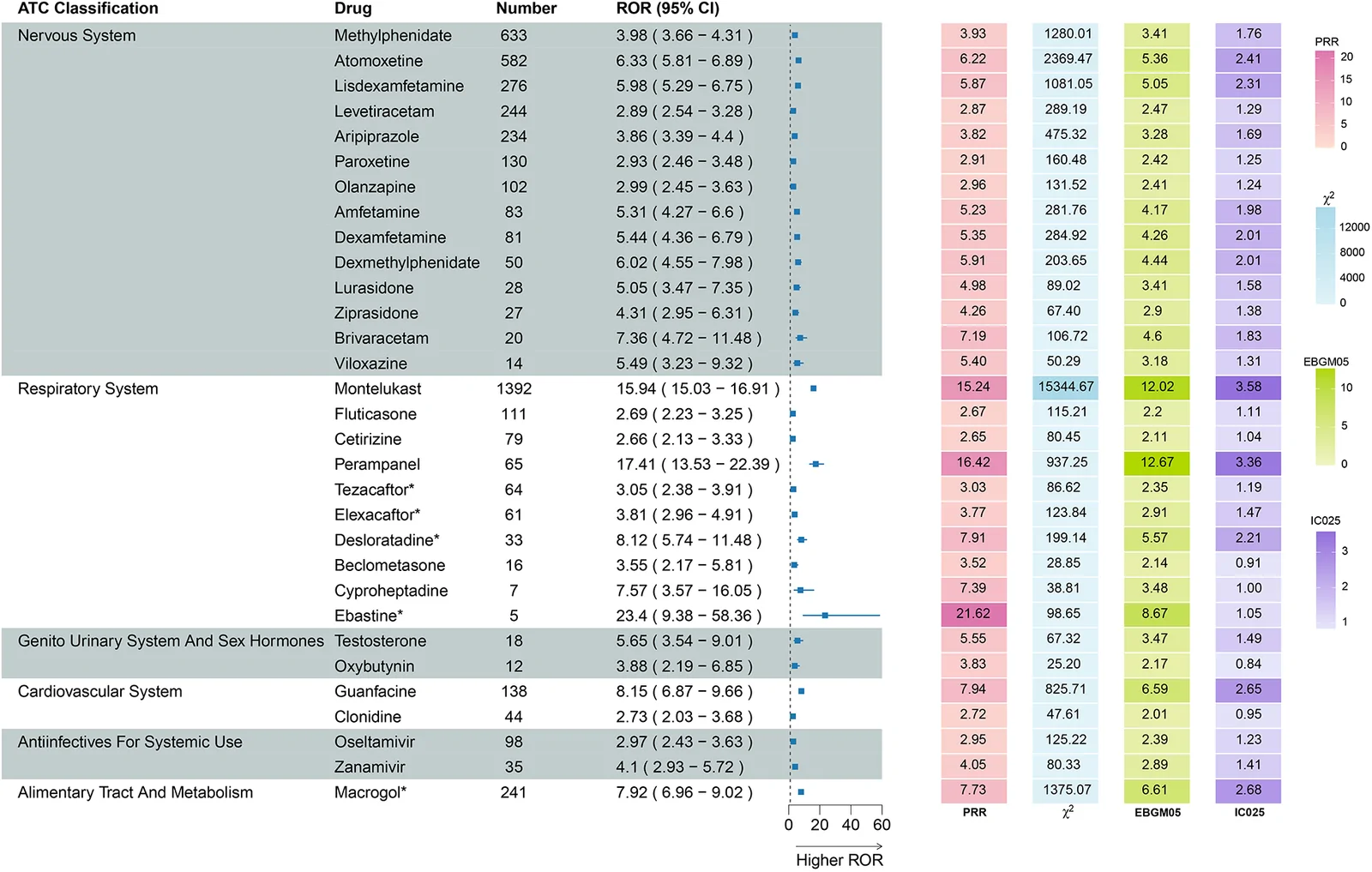
Signal values of 31 drugs with positive signals for aggression in different anatomical therapeutic chemical (ATC) categories. The forest plot ranks drugs by case count and displays the reporting odds ratio (ROR) with 95% confidence interval. The adjacent heatmap provides a multi-metric assessment of signal strength using the proportional reporting ratio (PRR), chi-squared (*χ*^2^), the lower limit of the 95% confidence interval for the Empirical Bayes Geometric Mean (EBGM05), and the lower end of the 95% credibility interval for the Information Component (IC025). *Adverse events related to aggression were not reported in the drug's prescribing information.

**Figure 4:**
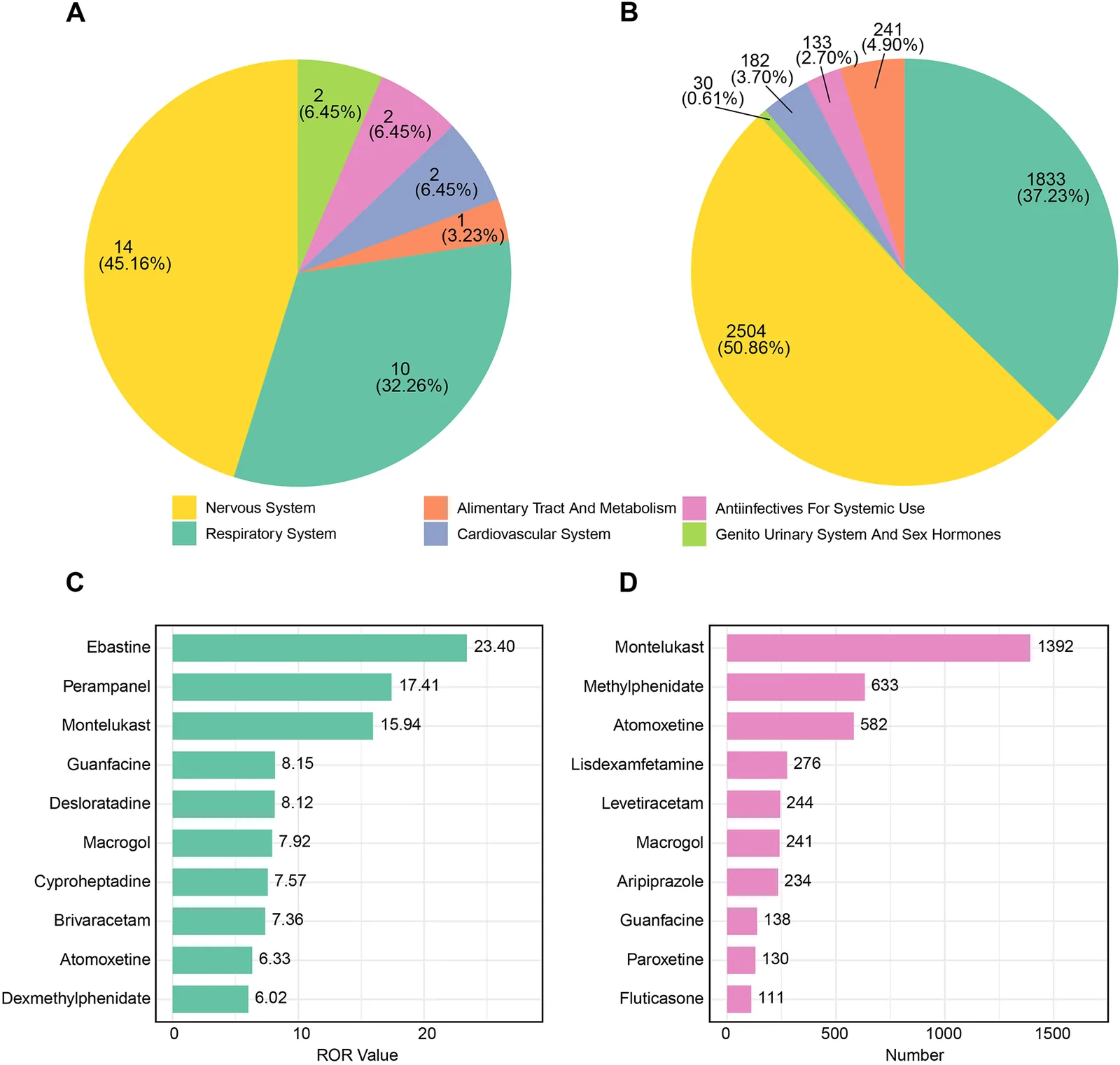
Classification and ranking of 31 drugs with positive disproportionality signals for aggression in a pediatric population. **(A)** Drug distribution by ATC class; **(B)** Report distribution by ATC class; **(C)** Top 10 drugs ranked by reporting odds ratio (ROR); **(D)** Top 10 drugs ranked by reported case count.

Thirty-one drugs met criteria for positive signals across all four disproportionality algorithms, representing 4,923 reports (Figure 3; Supplementary Table S3). Signals for tezacaftor and elexacaftor (CFTR modulators), macrogol, desloratadine, and ebastine were identified despite the absence of specific FDA label warnings for aggression; notably, ebastine remains unapproved by the FDA. Nervous system agents (14 drugs, 45.16%) and respiratory system agents (10 drugs, 32.26%) predominated by drug count (Figure 4A). In terms of report counts, the top two ATC categories were Nervous System (2,504 reports; 50.86%) and Respiratory System (1,833 reports; 37.23%), collectively representing 88.09% of the reports associated with positive signals (Figure 4B). The highest RORs were observed for ebastine (23.40), perampanel (17.41), and montelukast (15.94) (Figure 4C). By case volume, montelukast ranked first (*N* = 1,392), followed by methylphenidate (*N* = 633) and atomoxetine (*N* = 582) (Figure 4D). In sensitivity analysis restricted to non-U.S. reports (Supplementary Table S4), the signal for ebastine persisted and was slightly stronger (ROR: 25.23, 95% CI: 10.11–62.93; PRR: 23.31, *χ*^2^: 106.95; EBGM: 23.31, EBGM05: 10.85; IC: 4.54, IC025: 0.99), remaining positive across all algorithms.

### Age-related differences in aRORs

**Figure 5:**
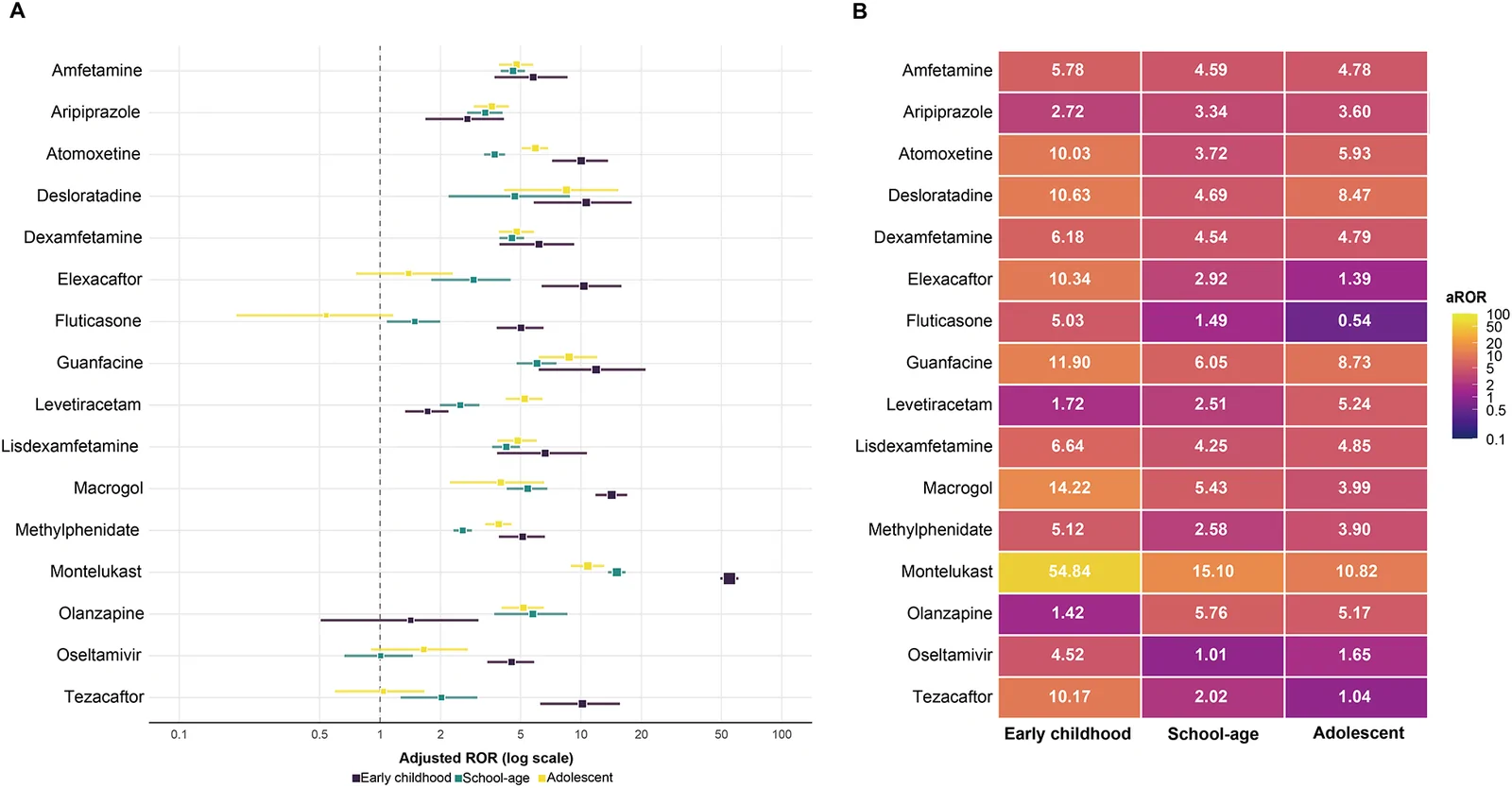
Age-stratified analysis of aggression signals for 16 drugs in pediatric patients. **(A)** Forest plot of adjusted reporting odds ratios (aRORs) with 95% confidence intervals across three age groups: early childhood (<6 years), school-age (≥6 to <12 years), and adolescence (≥12 to <18 years); **(B)** Heatmap visualization of the aROR signals.

Among 16 drugs meeting sample size criteria, most displayed highest aRORs in early childhood (*e.g.*, montelukast: aROR: 54.84, 95% CI: 49.39–60.86; macrogol: aROR: 14.22, 95% CI: 11.81–17.03), decreasing with age (Figure 5). Exceptions included levetiracetam, which peaked in adolescence (aROR: 5.24, 95% CI: 4.21–6.44), and olanzapine, with comparably elevated risks in school-age and adolescent groups.

**Figure 6:**
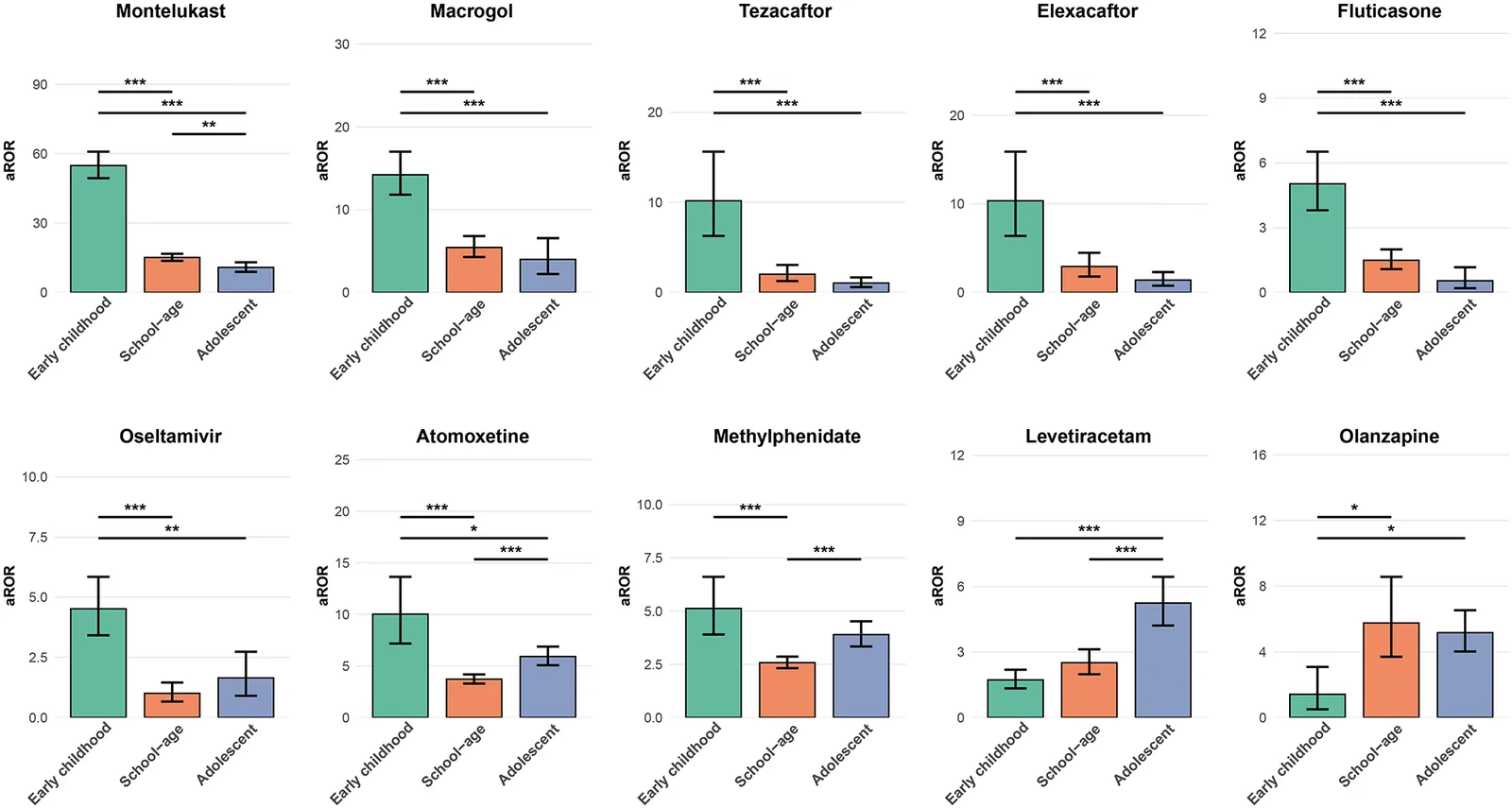
Multiple comparison-adjusted pairwise testing identified statistically significant, age-related differences in adjusted reporting odds ratios (aRORs) for 10 drugs. Significance levels are indicated as follows: *p < 0.05, **p < 0.01, ***p < 0.001 (Holm-adjusted).

Holm-adjusted pairwise comparisons identified significant age differences for 10 drugs (Figure 6), most commonly higher risk in early childhood vs. older strata. The most common pattern was a significantly elevated aROR in early childhood (<6 years) compared to older age groups, which was particularly notable for montelukast, macrogol, the elexacaftor/tezacaftor combination, fluticasone, and oseltamivir. In contrast, levetiracetam was associated with the highest risk in adolescents (≥12 to <18 years). For atomoxetine and methylphenidate, significantly elevated aRORs were observed in both early childhood and adolescence when compared with school-age children. Conversely, olanzapine showed an inverse relationship, with significantly greater risks in school-age children and adolescents than in the early childhood group.

### Sex-related differences in aRORs

**Figure 7:**
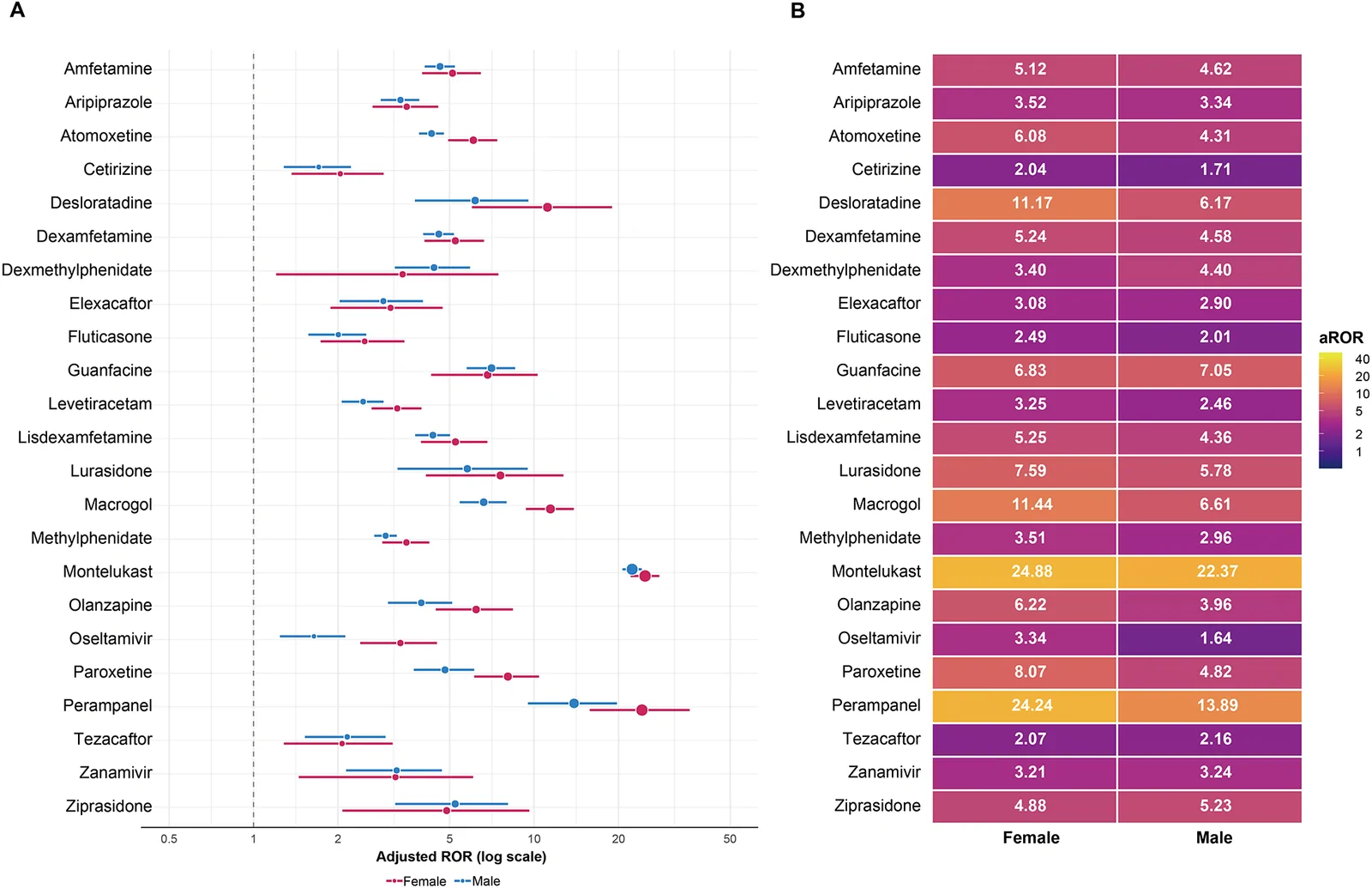
Sex-stratified analysis of aggression signals for 23 drugs in pediatric patients. **(A)** Forest plot of sex-specific adjusted reporting odds ratios (aRORs) with 95% confidence intervals, comparing females vs. males; **(B)** Heatmap visualization of the aROR signals.

**Figure 8:**
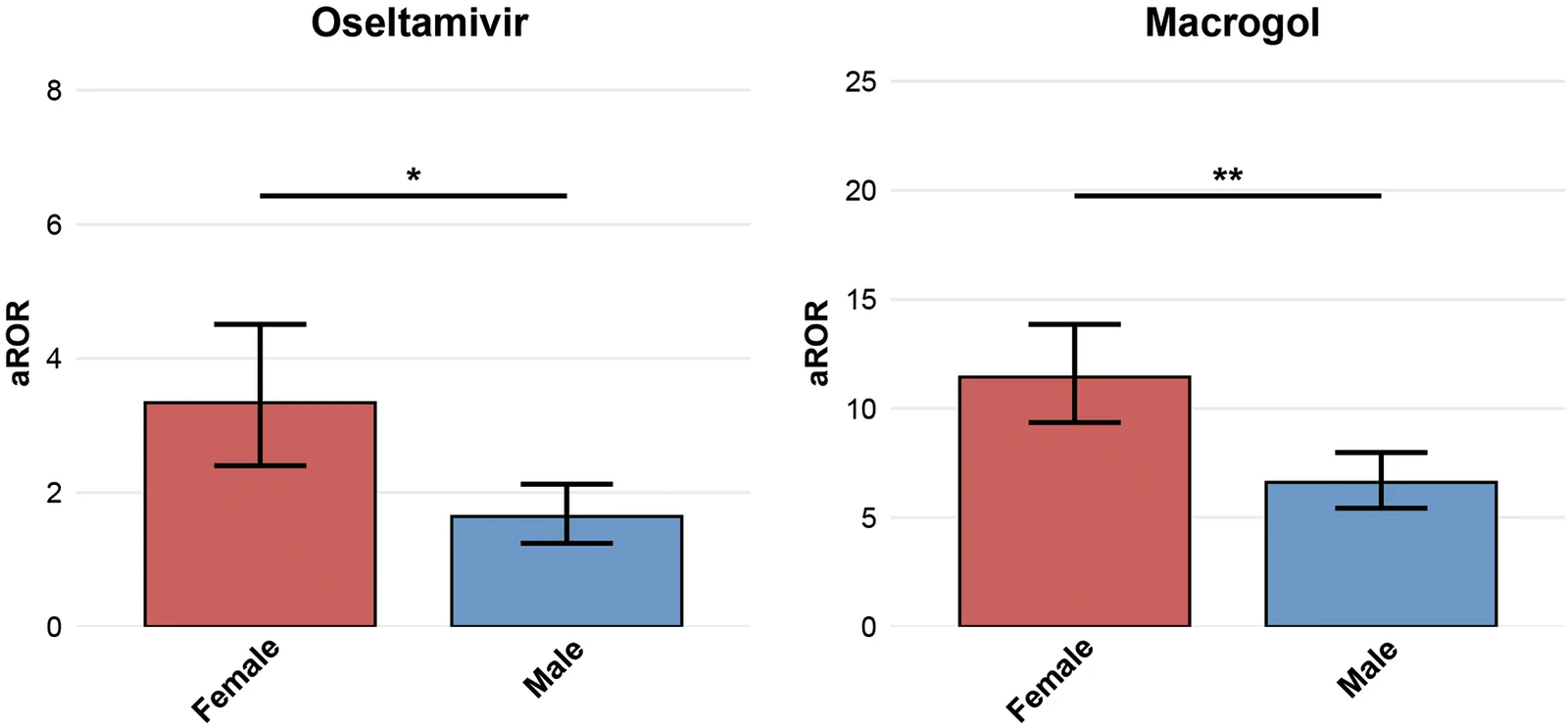
Multiple comparison-adjusted testing identified statistically significant sex differences in adjusted reporting odds ratios (aRORs) for 2 drugs. Significance levels are indicated as follows: *p < 0.05, **p < 0.01 (Holm-adjusted).

The analysis of sex-based differences was limited to 23 of the initial 31 drugs that satisfied the predefined sample size criteria. Examination of these 23 pediatric drugs indicated a general trend toward elevated adjusted reporting odds ratios (aRORs) for aggression in females relative to males for the majority of agents (Figure 7). After Holm adjustment, significant sex differences were confirmed for macrogol (female aROR: 11.44, 95% CI: 9.36–13.86 vs. male 6.61, 95% CI: 5.43–7.98) and oseltamivir (female aROR: 3.34, 95% CI: 2.40–4.51 vs. male 1.64, 95% CI: 1.24–2.12) (Figure 8).

### Time to onset (TTO) analysis

**Figure 9:**
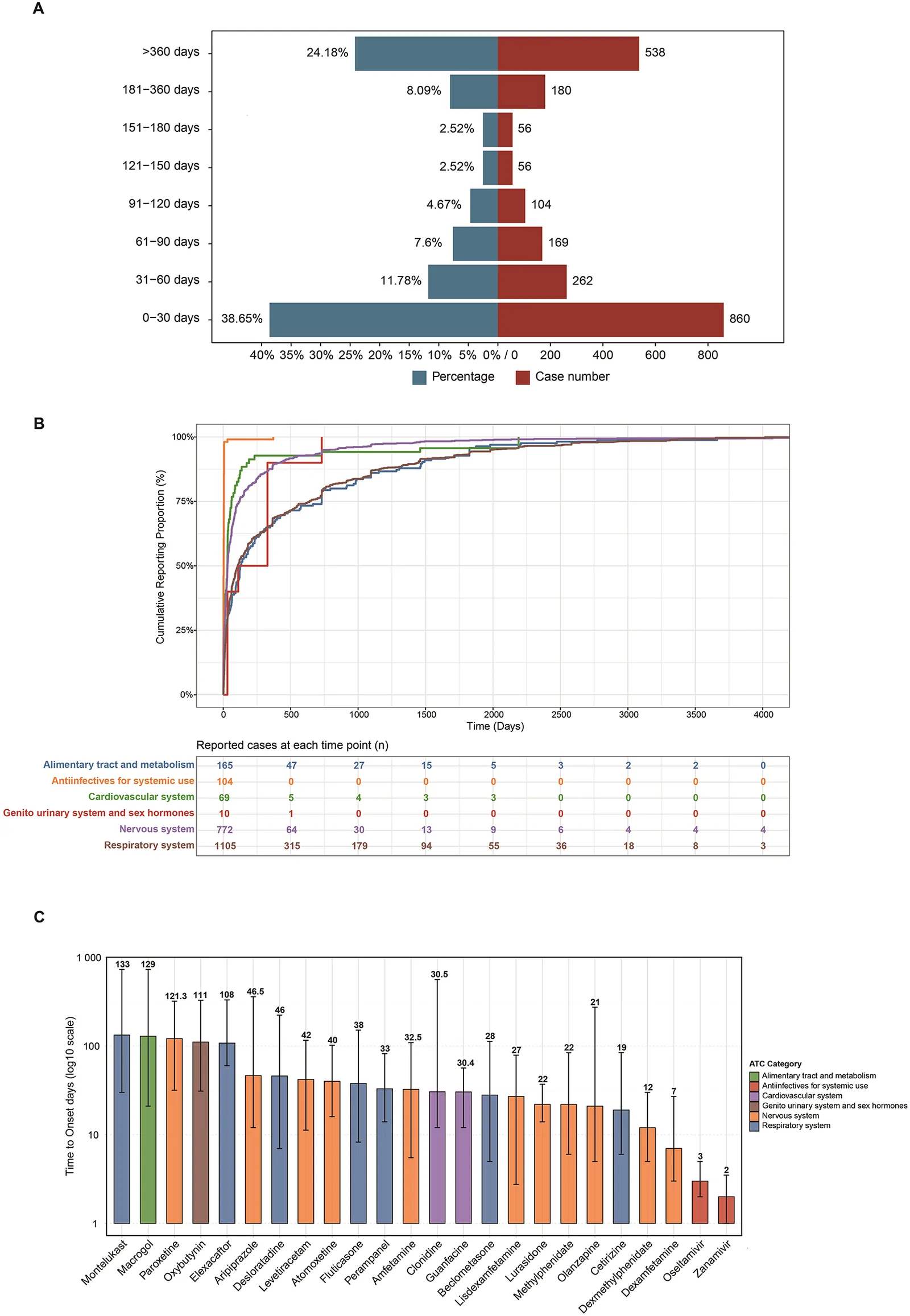
Time-to-onset analysis of drug-related aggression in pediatric patients. **(A)** TTO distribution across 31 drugs (*n* = 2,225 cases); **(B)** Cumulative reporting timeline of drug-related aggression based on different ATC classifications; **(C)** TTO distribution for 24 drugs (≥5 cases each) across ATC classes.

The time-to-onset (TTO) of aggressive behavior was analyzed for the 31 drugs with positive signals, using data from 2,225 pediatric reports with valid TTO information. Of these, 860 cases (38.7%) occurred within 30 days, while 538 cases (24.2%) had a TTO exceeding one year. The overall TTO distribution is illustrated in Figure 9A.

Cumulative reporting proportion curves, stratified by Anatomical Therapeutic Chemical (ATC) classification, highlighted distinct temporal patterns (Figure 9B). For instance, anti-infectives for systemic use showed a rapid initial rise followed by stabilization, indicating quick onset. Conversely, drugs from other categories, such as nervous system and respiratory system agents, displayed more gradual and varied progressions, suggesting delayed or protracted onset.

For the 24 drugs with at least five cases, median TTO varied considerably across ATC classes (Figure 9C). Anti-infectives had the shortest medians: zanamivir (2 days) and oseltamivir (3 days). Among respiratory agents, durations ranged from shorter medians like cetirizine (19 days), beclometasone (28 days), fluticasone (38 days), and desloratadine (46 days) to longer ones such as elexacaftor (108 days) and montelukast (133 days). Nervous system drugs showed medians from 7 days (dexamfetamine) to 121.3 days (paroxetine). Cardiovascular agents included guanfacine (30.4 days) and clonidine (30.5 days). In the alimentary tract and metabolism category, macrogol had a median of 129 days, while oxybutynin (genito-urinary system and sex hormones) was 111 days.

## Discussion

Prior research on drug-induced aggression in pediatric populations has predominantly relied on randomized controlled trials (RCTs) and small-scale cohort studies, which often overlook real-world variability and subgroup-specific risks, such as age- and sex-related differences (17, 18). These limitations include restricted sample sizes, short follow-up periods, selective enrollment criteria that exclude vulnerable subgroups (*e.g.*, preschoolers or those with comorbidities), and underrepresentation of rare or delayed neuropsychiatric adverse events. This study addresses these gaps by utilizing the FAERS database (January 2004—June 2025) to identify pharmacovigilance signals for aggression, while examining demographic disparities, temporal patterns, and time-to-onset (TTO) profiles in a substantial pediatric cohort.

Our analysis of 7,959 reports revealed distinct characteristics regarding patient demographics and reporting dynamics. Regarding sex distribution, males accounted for the majority (67.75%) compared with females (30.61%), and this sex disparity was consistent across all age strata. This pattern aligns with established evidence of increased vulnerability to aggressive behaviors in boys, potentially mediated by hormonal influences, genetic factors, and sex-specific neurobiological development (19, 20). Annual reporting trends peaked in 2019, followed by a decline from 2020 to 2022. This overall pattern was particularly pronounced for montelukast, the drug with the highest case volume in our dataset (*N* = 1,392), warranting a closer examination of its temporal reporting dynamics. The temporal analysis of montelukast reports revealed a distinct peak in 2019 (*N* = 133), followed by a progressive decline through 2022 (Supplementary Figure S1). This trend likely represents a variant of the “Weber effect” or 'stimulated reporting,’ where reporting frequency is driven by heightened external scrutiny rather than an actual change in the drug's safety profile (21, 22). Specifically, the surge in 2019 aligns with the FDA's Joint Advisory Committee meeting in September 2019, which conducted a high-profile review of montelukast's neuropsychiatric risks (23, 24). The resulting media coverage likely prompted caregivers and clinicians to retrospectively report adverse events. Following the formal Boxed Warning in March 2020, we observed a downward trend (*N* = 127 in 2020 to *N* = 56 in 2022) (25). This decline is consistent with recent drug utilization data showing that pediatric montelukast prescriptions fell from roughly 2.3 million users in 2018 to 1.6 million in 2022—a 30% reduction in the exposed population (26). Furthermore, the COVID-19 pandemic may have acted as a secondary confounder, as social distancing reduced allergen exposure and healthcare-seeking behavior, further suppressing the volume of spontaneous AE reports during 2020–2021 (27). Notably, the total number of pediatric FAERS reports displayed a closely parallel temporal pattern, with a decline during 2020–2021 followed by subsequent recovery (Supplementary Figure S2). This similarity suggests that the observed reduction in aggression-related reports primarily reflects broader under-reporting artifacts associated with the pandemic, rather than a genuine decrease in the incidence of aggression as an adverse event. This trend aligns with pandemic-related disruptions, such as diminished healthcare-seeking behavior and reduced non-COVID-related reporting activity. Similar declines in overall spontaneous ADR reporting during the early phases of the COVID-19 pandemic have been widely documented in FAERS and other global databases (27).

This large-scale pharmacovigilance analysis identified 31 drugs with robust disproportionality signals for aggression in pediatric patients, predominantly nervous system and respiratory agents. This distribution aligns with the therapeutic landscape for common pediatric conditions such as attention-deficit/hyperactivity disorder (ADHD) and asthma, where these classes are frequently prescribed, potentially heightening the risk of neuropsychiatric adverse events (AEs) in vulnerable young patients (28). Montelukast, exhibiting one of the strongest signals (ROR = 15.94; 95% CI: 15.03–16.91) and the highest report volume (*n* = 1,392), has been linked to neuropsychiatric effects, including aggression and irritability, with evidence suggesting onset within days of initiation and heightened susceptibility in children under 6 years (29, 30). This corroborates FDA-issued warnings for montelukast regarding serious mental health risks, emphasizing the need for targeted monitoring in pediatric asthma management. Notably, several high-signal drugs do not include specific mentions of aggression or closely related behavioral events in their FDA-approved prescribing information (tezacaftor, elexacaftor, desloratadine, macrogol) or lack any U.S. prescribing information due to non-approval (ebastine) (31). Furthermore, there is minimal evidence linking ebastine to aggressive behavior, with potential neuropsychiatric effects like abnormal behavior noted only in overdose contexts rather than therapeutic use (32). Although desloratadine, a second-generation antihistamine, is typically associated with minimal central nervous system side effects, pharmacovigilance signals from the WHO VigiBase database and Uppsala Monitoring Centre indicate an association with aggressive reactions in children (33). Furthermore, a Dutch pharmacovigilance study analyzing adverse drug reactions to systemic antihistamines in children from 1991 to 2014 identified milder neuropsychiatric effects, including aggression, agitation, and hyperactivity, associated with second-generation agents such as desloratadine (34). Tezacaftor and elexacaftor, components of CFTR modulator combinations (*e.g.*, elexacaftor/tezacaftor/ivacaftor) used in cystic fibrosis treatment (35), have come under recent scrutiny for potential mental health side effects. As of 2025, FDA labels for Trikafta do not include specific warnings for aggression but note “altered mental status” and “mental changes” as symptoms of liver injury (36). In contrast, EMA labels for Kaftrio explicitly mention depression (including suicidal ideation), anxiety, emotional discomfort, and behavioral changes, particularly in young children, with recommendations for monitoring (37). A French multicenter cohort study observed sudden behavioral changes, such as aggressive and oppositional behavior, in 47% of 197 preschool-aged children (aged 2–5 years) within the first month of elexacaftor–tezacaftor–ivacaftor (ETI) initiation (38). Macrogol (PEG), a commonly used osmotic laxative in children for constipation and bowel preparation, lacks neuropsychiatric warnings—including aggression—in standard labeling. In our analysis, PEG exhibited a positive signal for aggression. However, PEG is generally considered to have poor systemic absorption (<1.6%) and acts locally (39). Reported neuropsychiatric events in pediatric patients remain without an established direct causal link (40). These may instead involve indirect mechanisms, such as alterations in the gut microbiome, mild electrolyte imbalances with prolonged use, or confounding by underlying constipation severity (41, 42). Notably, our identified median time-to-onset (129 days) aligns with potential cumulative or indirect effects rather than acute systemic exposure.

Age-stratified results revealed markedly elevated risks in early childhood for most drugs, consistent with developmental immaturity and pharmacokinetic differences that heighten susceptibility to neuropsychiatric adverse reactions in younger patients (43). Notably, drugs such as montelukast, macrogol, elexacaftor, tezacaftor, fluticasone, atomoxetine, and oseltamivir exhibited higher aRORs in early childhood (<6 years). Montelukast exhibited the most pronounced early-childhood signal, contrasting with lower adolescent risk (29, 44). Notably, levetiracetam-associated aggression (aRORs) peaked in adolescence (≥12 to <18 years) in our analysis, contrasting with early childhood peaks observed for other anti-seizure medications (45, 46). While behavioral side effects, including the well-described “Keppra rage” phenotype (sudden, explosive aggression), are common across pediatric ages, this age-specific vulnerability may be influenced by several factors: (1) pharmacokinetic variability from rapid growth and frequent dose titrations; (2) pubertal hormonal shifts potentially synergizing with SV2A modulation to heighten impulsivity (47); and (3) unique psychosocial stressors in adolescents, such as academic demands and peer pressures (48–50). Given the limited and mixed evidence in the literature regarding age-dependent patterns of the “Keppra rage” phenotype, these findings support more cautious dose titration schedules and vigilant behavioral monitoring specifically in the adolescent population. Further prospective studies are warranted to clarify these age-related differences. Clinically, these age-specific signals advocate for personalized prescribing in pediatrics, with heightened vigilance for aggressive behaviors in younger children prescribed respiratory agents like montelukast or ADHD medication, though causal links require further prospective studies.

Sex differences showed a predominant pattern of higher aRORs in females, reaching statistical significance for macrogol and oseltamivir after adjustment. This may reflect sex-specific pharmacodynamic or hormonal influences, or reporting bias, as females often exhibit greater vulnerability to neuropsychiatric drug reactions. The female predominance with oseltamivir aligns with earlier reports of abnormal behavior during influenza treatment, potentially amplified by sex-dependent immune responses (51, 52).

The notably short median time-to-onset (2–3 days) for anti-infectives warrants particular attention. Although certain anti-infectives (*e.g.*, cefepime, carbapenems) are known to cause rapid-onset neuropsychiatric events such as delirium or encephalopathy (53, 54), the brevity observed here is more plausibly attributable to confounding by indication. Anti-infectives are typically prescribed for acute febrile illnesses in children, and high fever is a well-established trigger of pediatric delirium, manifesting as acute confusion, agitation, irritability, and behavioral changes that closely resemble aggression (55, 56). These symptoms typically resolve rapidly upon fever control or antipyretic treatment, consistent with the short TTO in our analysis. This illustrates a classic limitation of spontaneous reporting databases such as FAERS, where disease-related features (*e.g.*, fever or infection severity) can confound apparent drug-event associations, especially for short-onset signals in symptomatic indications. Future studies using clinical context, active comparators, or linked electronic health record data could help differentiate direct drug effects from underlying illness manifestations.

This study, like all pharmacovigilance analyses based on the FAERS database, is subject to several inherent limitations that require cautious interpretation of the identified signals (57). First, spontaneous reporting systems such as FAERS suffer from substantial underreporting—the so-called “tip of the iceberg” phenomenon—which likely results in an underestimation of the true incidence of drug-related aggression. Additionally, the absence of reliable denominator data (*e.g.*, total exposed population) precludes calculation of absolute risks or incidence rates. Consequently, disproportionality measures such as ROR and PRR should be viewed strictly as indicators of disproportionate reporting and potential signals, rather than as measures of true causal incidence (58). A major methodological challenge is confounding by indication. For example, in signals involving ADHD medications (*e.g.*, methylphenidate), the underlying condition itself is strongly associated with impulsivity, behavioral dysregulation, and aggression. The observed associations may therefore partly reflect the clinical features of the treated population rather than a direct adverse effect of the drug. This distinction is difficult to resolve in spontaneous reports due to the lack of detailed clinical context, and future studies employing active comparator designs or longitudinal electronic health record data will be essential to better disentangle these effects (59). Furthermore, despite adjustment for age and sex, residual confounding from polypharmacy and complex comorbidities persists as a significant concern. Pediatric patients frequently receive multiple concomitant medications, complicating attribution of aggressive behavior to any single suspect drug. Temporal biases, such as stimulated reporting (the “Weber effect”) following high-profile regulatory actions (*e.g.*, the 2020 FDA Boxed Warning for montelukast), may also inflate associations for certain drugs. In addition, the predominantly U.S.-centric dataset (56.51% of reports) may introduce geographic bias, limiting the generalizability of findings to non-U.S. populations or regions where reporting patterns or drug utilization differ (60). A notable limitation stemming from this U.S. dominance is the potential for geographic reporting biases, as differences in reporting practices, regulatory environments, or “clumping” of reports from specific countries may inflate signals for drugs primarily reported from non-U.S. sources (61). For instance, ebastine reports were exclusively from non-U.S. sources, potentially leading to denominator mismatch against a U.S.-heavy background. However, our sensitivity analysis restricting to non-U.S. reports confirmed a persistent positive signal for ebastine (ROR: 25.23, 95% CI: 10.11–62.93), suggesting that the association is not solely an artifact of geographic bias. This finding aligns with prior case reports of ebastine-associated behavioral changes in children (32), but underscores the need for caution in interpreting signals for internationally variable drugs. Future pharmacovigilance studies should routinely incorporate geographic stratification or linkage with prescription data to mitigate such confounders (17, 59). An inherent limitation is the lack of granular detail within the “Other Serious (Important Medical Event)” category, which accounted for 45.80% of cases. This standard FAERS outcome term denotes medically important events that do not meet criteria for other serious categories (*e.g.*, hospitalization). The absence of sub-classification for healthcare utilization, such as emergency room visits or outpatient management, prevents a more precise characterization of clinical severity. However, this does not compromise the validity of our primary disproportionality signals, as outcome data were used solely for descriptive purposes. Despite these constraints, the large sample size of FAERS enhances statistical power for detecting rare events. Overall, these limitations underscore the need for cautious, hypothesis-generating interpretation of the pharmacovigilance signals identified in this study.

Future investigations should employ prospective cohorts or randomized trials with global representation to confirm causality and explore mechanisms, such as neuroimaging for neurodevelopmental impacts or pharmacokinetic studies in subgroups (62). Longitudinal designs could assess long-term outcomes and intervention efficacy, addressing current gaps in pediatric safety monitoring. Furthermore, integrating pharmacovigilance signals and pediatric drug safety monitoring into the broader digital health ecosystem could enhance early detection, reporting, and management of neuropsychiatric adverse drug reactions in children—particularly during and after public health disruptions. Tools such as telemedicine, remote monitoring, and real-world data platforms offer promising opportunities for improved surveillance and timely interventions (63). Additionally, digitally enabled care models, including telemedicine, can improve the continuity, efficiency, and resilience of pediatric chronic disease management, thereby strengthening the translational relevance of these findings in evolving, technology-supported healthcare practices (64).

## Conclusions

In conclusion, this FAERS-based pharmacovigilance study reveals robust signals linking multiple drugs to aggression in pediatric patients, demonstrating significant age- and sex-related disparities, with heightened risks primarily for nervous and respiratory system agents. These findings underscore the need for enhanced clinical vigilance, timely label updates, and targeted risk-mitigation strategies to mitigate neuropsychiatric adverse events in vulnerable subgroups, emphasizing the importance of age- and sex-specific considerations in pediatric drug safety.

## Data Availability

The raw data supporting the conclusions of this article will be made available by the authors, upon request and without undue reservation.

## Supplementary material

Supplementary Figure S1 Annual trends of drug-related aggression reports in pediatric patients in the FAERS database from 2004 to 2025 (Q1–Q2). Annual trends of reported aggression cases, with vertical lines indicating the Joint Meeting of the Pediatric Advisory Committee and the Drug Safety and Risk Management Advisory Committee (PAC-DSaRM) in September 2019 (dashed) and the March 2020 FDA Boxed Warning (solid). The 2019 peak in montelukast reports reflects “stimulated reporting” driven by these regulatory milestones.

Supplementary Figure S2 Comparative annual trends of drug-related aggression reports versus total pediatric reports in the FAERS database (2004–2025 (Q1–Q2)). Annual number of drug-associated aggression reports in pediatric patients (blue line, left y-axis) and total number of all pediatric FAERS reports (green line, right y-axis). Data points represent absolute report counts per year; 2025 data are limited to the first two quarters (Q1–Q2).

## References

[1] Loeber R Burke JD Lahey BB Winters A Zera M. Oppositional defiant and conduct disorder: a review of the past 10 years, part I. J Am Acad Child Adolesc Psychiatry. (2000) 39(12):1468–84. 10.1097/00004583-200012000-00007 11128323

[2] Tremblay RE. Developmental origins of disruptive behaviour problems: the ‘original sin’ hypothesis, epigenetics and their consequences for prevention. J Child Psychol Psychiatry. (2010) 51(4):341–67. 10.1111/j.1469-7610.2010.02211.x 20146751

[3] Moore TJ Glenmullen J Furberg CD. Prescription drugs associated with reports of violence towards others. PLoS One. (2010) 5(12):e15337. 10.1371/journal.pone.0015337 21179515 PMC3002271

[4] Luft MJ Lamy M DelBello MP McNamara RK Strawn JR. Antidepressant-Induced activation in children and adolescents: risk, recognition and management. Curr Probl Pediatr Adolesc Health Care. (2018) 48(2):50–62. 10.1016/j.cppeds.2017.12.001 29358037 PMC5828909

[5] Jane Garland E Kutcher S Virani A Elbe D. Update on the use of SSRIs and SNRIs with children and adolescents in clinical practice. J Can Acad Child Adolesc Psychiatry. (2016) 25(1):4–10. 27047551 PMC4791100

[6] Cortese S Holtmann M Banaschewski T Buitelaar J Coghill D Danckaerts M. Practitioner review: current best practice in the management of adverse events during treatment with ADHD medications in children and adolescents. J Child Psychol Psychiatry. (2013) 54(3):227–46. 10.1111/jcpp.12036 23294014

[7] Warrer P Thomsen PH Dalsgaard S Hansen EH Aagaard L Wallach Kildemoes H. Switch in therapy from methylphenidate to atomoxetine in children and adolescents with attention-deficit/hyperactivity disorder: an analysis of patient records. J Child Adolesc Psychopharmacol. (2016) 26(4):354–61. 10.1089/cap.2015.0060 26891424 PMC4876536

[8] Polanczyk GV Salum GA Sugaya LS Caye A Rohde LA. Annual research review: a meta-analysis of the worldwide prevalence of mental disorders in children and adolescents. J Child Psychol Psychiatry. (2015) 56(3):345–65. 10.1111/jcpp.12381 25649325

[9] Turner EH Matthews AM Linardatos E Tell RA Rosenthal R. Selective publication of antidepressant trials and its influence on apparent efficacy. N Engl J Med. (2008) 358(3):252–60. 10.1056/NEJMsa065779 18199864

[10] Sakaeda T Tamon A Kadoyama K Okuno Y. Data mining of the public version of the FDA adverse event reporting system. Int J Med Sci. (2013) 10(7):796–803. 10.7150/ijms.6048 23794943 PMC3689877

[11] Montastruc JL Sommet A Bagheri H Lapeyre-Mestre M. Benefits and strengths of the disproportionality analysis for identification of adverse drug reactions in a pharmacovigilance database. Br J Clin Pharmacol. (2011) 72(6):905–8. 10.1111/j.1365-2125.2011.04037.x 21658092 PMC3244636

[12] Glickman ME Rao SR Schultz MR. False discovery rate control is a recommended alternative to Bonferroni-type adjustments in health studies. J Clin Epidemiol. (2014) 67(8):850–7. 10.1016/j.jclinepi.2014.03.012 24831050

[13] Szarfman A Machado SG O'Neill RT. Use of screening algorithms and computer systems to efficiently signal higher-than-expected combinations of drugs and events in the US FDA’s spontaneous reports database. Drug Saf. (2002) 25(6):381–92. 10.2165/00002018-200225060-00001 12071774

[14] Bate A Evans SJ. Quantitative signal detection using spontaneous ADR reporting. Pharmacoepidemiol Drug Saf. (2009) 18(6):427–36. 10.1002/pds.1742 19358225

[15] Evans SJ Waller PC Davis S. Use of proportional reporting ratios (PRRs) for signal generation from spontaneous adverse drug reaction reports. Pharmacoepidemiol Drug Saf. (2001) 10(6):483–6. 10.1002/pds.677 11828828

[16] Rothman KJ Lanes S Sacks ST. The reporting odds ratio and its advantages over the proportional reporting ratio. Pharmacoepidemiol Drug Saf. (2004) 13(8):519–23. 10.1002/pds.1001 15317031

[17] Elshafie S Zaghloul I Roberti AM. Pharmacovigilance in developing countries (part I): importance and challenges. Int J Clin Pharm. (2018) 40(4):758–63. 10.1007/s11096-017-0570-z 29248988

[18] Bellis JR Kirkham JJ Nunn AJ Pirmohamed M. Adverse drug reactions and off-label and unlicensed medicines in children: a prospective cohort study of unplanned admissions to a paediatric hospital. Br J Clin Pharmacol. (2014) 77(3):545–53. 10.1111/bcp.12222 23919928 PMC4371534

[19] Thijssen S Ringoot AP Wildeboer A Bakermans-Kranenburg MJ El Marroun H Hofman A. Brain morphology of childhood aggressive behavior: a multi-informant study in school-age children. Cogn Affect Behav Neurosci. (2015) 15(3):564–77. 10.3758/s13415-015-0344-9 25801924 PMC4526589

[20] Koyama E Kant T Takata A Kennedy JL Zai CC. Genetics of child aggression, a systematic review. Transl Psychiatry. (2024) 14(1):252. 10.1038/s41398-024-02870-7 38862490 PMC11167064

[21] Hoffman KB Dimbil M Erdman CB Tatonetti NP Overstreet BM. The weber effect and the United States food and drug administration’s adverse event reporting system (FAERS): analysis of sixty-two drugs approved from 2006 to 2010. Drug Saf. (2014) 37(4):283–94. 10.1007/s40264-014-0150-2 24643967 PMC3975089

[22] Arora A Jalali RK Vohora D. Relevance of the weber effect in contemporary pharmacovigilance of oncology drugs. Ther Clin Risk Manag. (2017) 13:1195–203. 10.2147/TCRM.S137144 28979130 PMC5602442

[23] Clarridge K Chin S Eworuke E Seymour S. A boxed warning for montelukast: the FDA perspective. J Allergy Clin Immunol Pract. (2021) 9(7):2638–41. 10.1016/j.jaip.2021.02.057 33744471

[24] U.S. Food and Drug Administration. September 27, 2019: Meeting of the Pediatric and Drug Safety and Risk Management Committees. Silver Spring, MD: U.S. Food and Drug Administration (2019).

[25] Abdelkader S Hendrix-Dicken AD Condren M. The impact of Montelukast’s black box warning on pediatric mental health adverse event reports. J Pediatr Pharmacol Ther. (2023) 28(8):704–9. 10.5863/1551-6776-28.8.704 38094668 PMC10715380

[26] Jewett CM Benjamin F.D.A. Warned of Mental Side Effects from Asthma Drug, Singulair. Few Were Told. In: *The New York Times*. (2024).

[27] Tsuchiya M Sakai T Okada N Funakoshi R Masuyama K Mano N. Changes in the trend and quality of adverse drug reaction reports in the Japanese adverse drug event report database and the impact of COVID-19-related reports. Int J Pharm Pract. (2025):riaf084. 10.1093/ijpp/riaf084 41014315

[28] Hales CM Kit BK Gu Q Ogden CL. Trends in prescription medication use among children and adolescents-United States, 1999–2014. JAMA. (2018) 319(19):2009–20. 10.1001/jama.2018.5690 29800213 PMC6583241

[29] Haarman MG van Hunsel F de Vries TW. Adverse drug reactions of montelukast in children and adults. Pharmacol Res Perspect. (2017) 5(5):e00341. 10.1002/prp2.341 28971612 PMC5625152

[30] Bian S Li L Wang Z Cui L Xu Y Guan K. Neuropsychiatric side reactions of leukotriene receptor antagonist, antihistamine, and inhaled corticosteroid: a real-world analysis of the food and drug administration (FDA) adverse event reporting system (FAERS). World Allergy Organ J. (2021) 14(10):100594. 10.1016/j.waojou.2021.100594 34659626 PMC8498094

[31] Rico S Antonijoan R Barbanoj M. Ebastine in the light of CONGA recommendations for the development of third-generation antihistamines. J Asthma Allergy. (2009) 2:73–92. 10.2147/jaa.s3108 21437146 PMC3048600

[32] Pecoraro L Paiola G Pietrobelli A. Ebastine overdose in a child. Clin Case Rep. (2017) 5(4):403–5. 10.1002/ccr3.845 28396756 PMC5378857

[33] Sandberg L. Desloratadine and aggressive reaction. WHO Pharmaceut Newsl. (2015) (6):25–30.

[34] de Vries TW van Hunsel F. Adverse drug reactions of systemic antihistamines in children in The Netherlands. Arch Dis Child. (2016) 101(10):968–70. 10.1136/archdischild-2015-310315 27091848

[35] Zemanick ET Taylor-Cousar JL Davies J Gibson RL Mall MA McKone EF. A phase 3 open-label study of elexacaftor/tezacaftor/ivacaftor in children 6 through 11 years of age with cystic fibrosis and at least one F508del allele. Am J Respir Crit Care Med. (2021) 203(12):1522–32. 10.1164/rccm.202102-0509OC 33734030 PMC8483230

[36] U.S. Food and Drug Administration. TRIKAFTA (Elexacaftor, Tezacaftor, and Ivacaftor Tablets; Ivacaftor Tablets) Prescribing Information. Silver Spring, MD: U.S. Food and Drug Administration (2025).

[37] European Medicines Agency. Kaftrio (Ivacaftor/Tezacaftor/Elexacaftor) EPAR Product Information. Amsterdam, Netherlands: European Medicines Agency (2025).

[38] Sermet-Gaudelus I Benaboud S Bui S Bihouee T Gautier S, group M-Cs. Behavioural and sleep issues after initiation of elexacaftor-tezacaftor-ivacaftor in preschool-age children with cystic fibrosis. Lancet. (2024) 404(10448):117–20. 10.1016/S0140-6736(24)01134-6 38950554

[39] Muller-Lissner S. Classification, pharmacology, and side-effects of common laxatives. Ital J Gastroenterol Hepatol. (1999) 31(3):S234–237. 10726225

[40] Hussain SZB-GJ Chogle A Bhuiyan MAN Hicks T Misra S. Probable neuropsychiatric toxicity of polyethylene glycol: roles of media, internet and the caregivers. GastroHep. (2019) 1(3):118–23. 10.1002/ygh2.336

[41] Kolosionek TJ Jiang RY Meleis MM Ebeling-Koning NE Surmaitis RM. Polyethylene glycol misuse causing acute renal failure and metabolic acidosis requiring dialysis: a case report. Cureus. (2024) 16(7):e65838. 10.7759/cureus.65838 39219942 PMC11363814

[42] Salman SS Williams KC Marte-Ortiz P Rumpf W Mashburn-Warren L Lauber CL. Polyethylene glycol 3350 changes stool consistency and the microbiome but not behavior of CD1 mice. J Pediatr Gastroenterol Nutr. (2021) 73(4):499–506. 10.1097/MPG.0000000000003222 34238825

[43] Lu H Rosenbaum S. Developmental pharmacokinetics in pediatric populations. J Pediatr Pharmacol Ther. (2014) 19(4):262–76. 10.5863/1551-6776-19.4.262 25762871 PMC4341411

[44] Benard B Bastien V Vinet B Yang R Krajinovic M Ducharme FM. Neuropsychiatric adverse drug reactions in children initiated on montelukast in real-life practice. Eur Respir J. (2017) 50(2):1700148. 10.1183/13993003.00148-2017 28818882

[45] Hosoya R Kitajima K Sogawa K Ikegami D Terajima T Kato H. Principal component analysis of antiseizure medication-induced hostility/aggression and factor analysis of levetiracetam using the food and drug administration adverse event reporting system. Epilepsy Res. (2025) 218:107626. 10.1016/j.eplepsyres.2025.107626 40694860

[46] Li J Zhong R Zhang F Guo Y. Psychiatric disorders associated with newer antiseizure medications: a real-world disproportionality analysis of FDA adverse event reporting system. Epilepsy Behav. (2025) 172:110722. 10.1016/j.yebeh.2025.110722 41016122

[47] Hansen CC Ljung H Brodtkorb E Reimers A. Mechanisms underlying aggressive behavior induced by antiepileptic drugs: focus on topiramate, levetiracetam, and perampanel. Behav Neurol. (2018) 2018:2064027. 10.1155/2018/2064027 30581496 PMC6276511

[48] Vatansever Pinar Z Sager SG Cimen ID Cag Y. The effect of levetiracetam and valproic acid treatment on anger and attention deficit hyperactivity disorder clinical features in children and adolescents with epilepsy: a prospective study. Paediatr Drugs. (2024) 26(6):753–65. 10.1007/s40272-024-00652-8 39331340

[49] Helmstaedter C Fritz NE Kockelmann E Kosanetzky N Elger CE. Positive and negative psychotropic effects of levetiracetam. Epilepsy Behav. (2008) 13(3):535–41. 10.1016/j.yebeh.2008.05.012 18583196

[50] Dhungel O Shrestha A Pathak P Sharma P. Levetiracetam-induced acute psychosis in an adolescent. Case Rep Psychiatry. (2023) 2023:5575900. 10.1155/2023/5575900 37745836 PMC10516691

[51] Toovey S Rayner C Prinssen E Chu T Donner B Thakrar B. Assessment of neuropsychiatric adverse events in influenza patients treated with oseltamivir: a comprehensive review. Drug Saf. (2008) 31(12):1097–114. 10.2165/0002018-200831120-00006 19026027

[52] Klein SL Hodgson A Robinson DP. Mechanisms of sex disparities in influenza pathogenesis. J Leukoc Biol. (2012) 92(1):67–73. 10.1189/jlb.0811427 22131346 PMC4046247

[53] Teng C Frei CR. Delirium associations with antibiotics: a pharmacovigilance study of the FDA adverse event reporting system (FAERS). Drugs Real World Outcomes. (2022) 9(1):23–9. 10.1007/s40801-021-00268-1 34275113 PMC8844315

[54] Li HT Lee CH Wu T Cheng MY Tseng WJ Chang CW. Clinical, electroencephalographic features and prognostic factors of cefepime-induced neurotoxicity: a retrospective study. Neurocrit Care. (2019) 31(2):329–37. 10.1007/s12028-019-00682-y 30756319

[55] Onoe S Nishigaki T. EEG Spectral analysis in children with febrile delirium. Brain Dev. (2004) 26(8):513–8. 10.1016/j.braindev.2004.02.004 15533652

[56] Augenstein JA Klein EJ Traube C. Delirium upon presentation to the pediatric emergency department: a case series. Pediatr Emerg Care. (2018) 34(8):e147–9. 10.1097/PEC.0000000000001175 28590990

[57] Al Dweik R Stacey D Kohen D Yaya S. Factors affecting patient reporting of adverse drug reactions: a systematic review. Br J Clin Pharmacol. (2017) 83(4):875–83. 10.1111/bcp.13159 27868226 PMC5346870

[58] Cutroneo PM Sartori D Tuccori M Crisafulli S Battini V Carnovale C. Conducting and interpreting disproportionality analyses derived from spontaneous reporting systems. Front Drug Saf Regul. (2023) 3:1323057. 10.3389/fdsfr.2023.1323057 40980108 PMC12443087

[59] Sendor R Sturmer T. Core concepts in pharmacoepidemiology: confounding by indication and the role of active comparators. Pharmacoepidemiol Drug Saf. (2022) 31(3):261–9. 10.1002/pds.5407 35019190 PMC9121653

[60] Ning W Huang X Li S Jiang L Reissmann DR Kreher D. Real-world sodium fluoride safety signals: dual-database pharmacovigilance. Int Dent J. (2026) 76(2):109423. 10.1016/j.identj.2026.109423 41650835 PMC12905758

[61] Marrero O Hung EY Hauben M. Seasonal and geographic variation in adverse event reporting. Drugs Real World Outcomes. (2016) 3(3):297–306. 10.1007/s40801-016-0081-6 27747826 PMC5042937

[62] Berkley JA Walson JL Gray G Russell F Bhutta Z Ashorn P. Strengthening the paediatric clinical trial ecosystem to better inform policy and programmes. Lancet Glob Health. (2025) 13(4):e732–9. 10.1016/S2214-109X(24)00511-4 40155110

[63] Tahmasbi F Toni E Javanmard Z Kheradbin N Nasiri S Sadoughi F. An overview of reviews on digital health interventions during COVID- 19 era: insights and lessons for future pandemics. Arch Public Health. (2025) 83(1):129. 10.1186/s13690-025-01590-8 40346715 PMC12063330

[64] Asadi H Toni E Ayatollahi H. Application of telemedicine technology for cardiovascular diseases management during the COVID-19 pandemic: a scoping review. Front Cardiovasc Med. (2024) 11:1397566. 10.3389/fcvm.2024.1397566 39188320 PMC11345180

