# Correction: Risk of drug-related aggression in pediatric populations: a pharmacovigilance analysis using the FAERS database

**DOI:** 10.3389/fped.2026.2030718

**Published:** 2026-10-26

**Authors:** Jing Chen, Lilan Zhao

**Affiliations:** 1 Department of Pharmacy, Fujian Children’s Hospital (Fujian Branch of Shanghai Children’s Medical Center), Fuzhou, China; 2 Department of Thoracic Surgery, Shengli Clinical Medical College of Fujian Medical University, Fujian Provincial Hospital, Fuzhou University Affiliated Provincial Hospital, Fuzhou, China

**Keywords:** disproportionality analysis, drug-related aggression, FAERS, pediatric populations, pharmacovigilance

An incorrect number was provided for “the National Natural Science Foundation of China”. The correct number is “82303374”.

The original version of this article has been updated.

