# Supplementary figures and images for: Risk of drug-related aggression in pediatric populations: a pharmacovigilance analysis using the FAERS database
Source: Front Pediatr. 2026 Jun 5;14:1803086. doi: 10.3389/fped.2026.1803086 (PMC13279699; doi:10.3389/fped.2026.1803086)

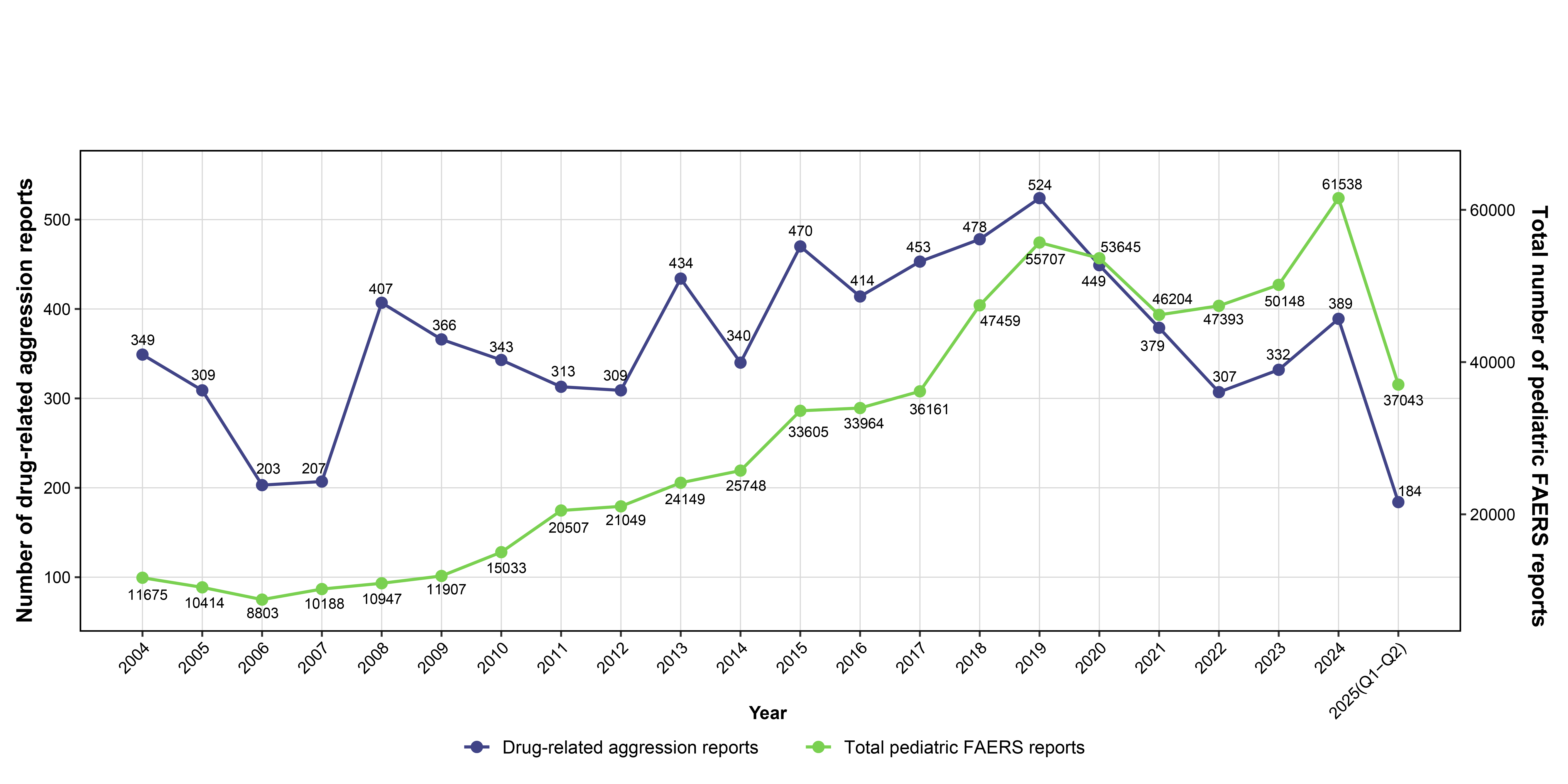

Supplement: Supplementary Figure S1 — Annual trends of drug-related aggression reports in pediatric patients in the FAERS database from 2004 to 2025 (Q1–Q2). Annual trends of reported aggression cases, with vertical lines indicating the Joint Meeting of the Pediatric Advisory Committee and the Drug Safety and Risk Management Advisory Committee (PAC-DSaRM) in September 2019 (dashed) and the March 2020 FDA Boxed Warning (solid). The 2019 peak in montelukast reports reflects “stimulated reporting” driven by these regulatory milestones. [file Image1.jpeg]

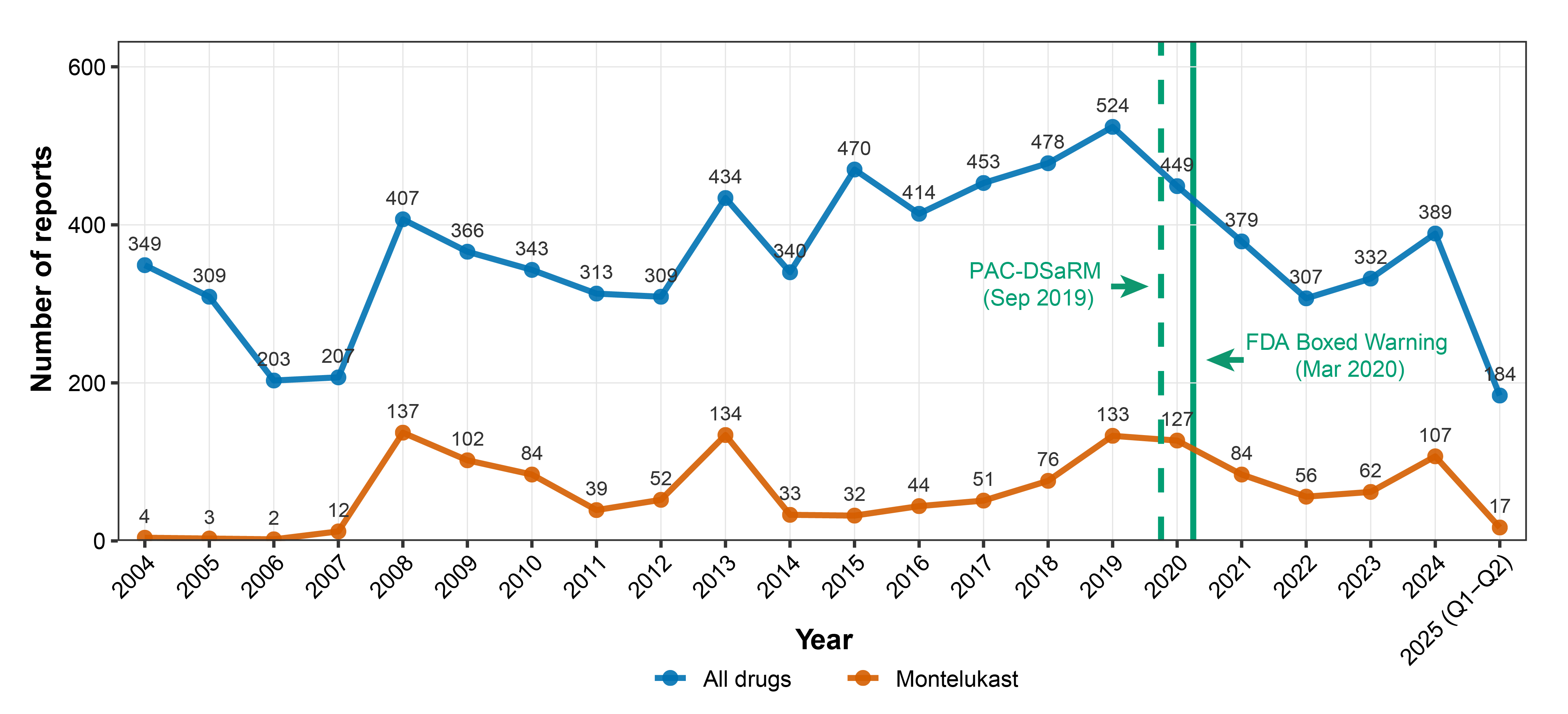

Supplement: Supplementary Figure S2 — Comparative annual trends of drug-related aggression reports versus total pediatric reports in the FAERS database (2004–2025 (Q1–Q2)). Annual number of drug-associated aggression reports in pediatric patients (blue line, left y-axis) and total number of all pediatric FAERS reports (green line, right y-axis). Data points represent absolute report counts per year; 2025 data are limited to the first two quarters (Q1–Q2). [file Image2.jpeg]
