# Supplementary material for: Risk of drug-related aggression in pediatric populations: a pharmacovigilance analysis using the FAERS database
Source: Front Pediatr. 2026 Jun 5;14:1803086. doi: 10.3389/fped.2026.1803086 (PMC13279699; doi:10.3389/fped.2026.1803086)
Supplement: Supplementary file 3 [file Table1.docx]

| **Supplementary Table 1. Contingency table for disproportionality analysis methods** | | | |
| --- | --- | --- | --- |
| Item | Target adverse events | All other adverse events | Total |
| Target drugs | a | b | a+b |
| All other drugs | c | d | c + d |
| Total | a+c | b+d | a+b + c + d |

**Notes:** A contingency table for the calculation formula of the proportion imbalance analysis.

**Supplementary Table 2. ROR, PRR, BCPNN, and MGPS methods, formulas, and thresholds**

| Algorithms | Equation | Criteria |
| --- | --- | --- |
| ROR | ROR=ad/cb  95%CI=eln(ROR)±1.96(1/a+1/b+1/c+1/d)^0.5 | lower limit of 95% CI>1,  a ≥ 3 |
| PRR | PRR=[a/(a+b)]/[c/(c+d)]  χ2=[(ad-bc)2(a+b+c+d)]/[(a+b)(c+d)(a+c)(b+d)] | PRR ≥ 2, χ2 ≥ 4, a ≥ 3 |
| BCPNN | IC=log2 [a (a+b+c+d)]/[(a+c)(a+b)]  95CI=eln(IC)±1.96(1/a+1/b+1/c+1/d)^0.5 | IC025>0 |
| MGPS | EBGM=a (a+b+c+d)/[(a+c)(a+b)]  EBGM05=95%CI=eln(EBGM)±1.96(1/a+1/b+1/c+1/d)^0.5 | EBGM05>2 |

**Notes:** Formulas for Four Pharmacovigilance Algorithms. Equation variables: a, number of reports involving both the target drug and the target adverse drug reaction; b, number of reports involving other adverse drug reactions associated with the target drug; c, number of reports involving the target adverse drug reaction associated with other drugs; d, number of reports involving other drugs and other adverse drug reactions.

**Abbreviation:** ROR, reporting odds ratio; PRR, proportional reporting ratio; BCPNN, Bayesian confidence propagation neural network; EBGM, empirical Bayes geometric mean; 95% CI, 95% confidence interval; χ², chi-squared; IC, information component; IC025, lower end of the 95% credibility interval for the IC; EBGM05, lower limit of the 95% confidence interval for the Empirical Bayes Geometric Mean.

**Supplementary Table 3. Sensitivity analysis of disproportionality signals for drug-related aggression among the 31 signal-positive drugs**

| **Drug Name** | **Number** | **ROR (95%CI)** | **PRR (95%CI)** | **PRR (**χ²) | **EBGM (EBGM05)** | **IC (IC025)** | ***p*** Value | **FDR​​** |
| --- | --- | --- | --- | --- | --- | --- | --- | --- |
| Montelukast | 1392 | 15.94 (15.03-16.91) | 15.24 (14.40-16.13) | 15.24 (15344.67) | 12.75 (12.02) | 3.67 (3.58) | <0.001 | <0.001 |
| Methylphenidate | 633 | 3.98 (3.66-4.31) | 3.93 (3.63-4.27) | 3.93 (1280.01) | 3.70 (3.41) | 1.89 (1.76) | <0.001 | <0.001 |
| Atomoxetine | 582 | 6.33 (5.81-6.89) | 6.22 (5.72-6.76) | 6.22 (2369.47) | 5.83 (5.36) | 2.54 (2.41) | <0.001 | <0.001 |
| Lisdexamfetamine | 276 | 5.98 (5.29-6.75) | 5.87 (5.21-6.61) | 5.87 (1081.05) | 5.70 (5.05) | 2.51 (2.31) | <0.001 | <0.001 |
| Levetiracetam | 244 | 2.89 (2.54-3.28) | 2.87 (2.53-3.26) | 2.87 (289.19) | 2.81 (2.47) | 1.49 (1.29) | <0.001 | <0.001 |
| Macrogol | 241 | 7.92 (6.96-9.02) | 7.73 (6.81-8.78) | 7.73 (1375.07) | 7.53 (6.61) | 2.91 (2.68) | <0.001 | <0.001 |
| Aripiprazole | 234 | 3.86 (3.39-4.40) | 3.82 (3.36-4.35) | 3.82 (475.32) | 3.74 (3.28) | 1.90 (1.69) | <0.001 | <0.001 |
| Guanfacine | 138 | 8.15 (6.87-9.66) | 7.94 (6.73-9.37) | 7.94 (825.71) | 7.82 (6.59) | 2.97 (2.65) | <0.001 | <0.001 |
| Paroxetine | 130 | 2.93 (2.46-3.48) | 2.91 (2.45-3.45) | 2.91 (160.48) | 2.88 (2.42) | 1.52 (1.25) | <0.001 | <0.001 |
| Fluticasone | 111 | 2.69 (2.23-3.25) | 2.67 (2.22-3.22) | 2.67 (115.21) | 2.65 (2.20) | 1.41 (1.11) | <0.001 | <0.001 |
| Olanzapine | 102 | 2.99 (2.45-3.63) | 2.96 (2.44-3.60) | 2.96 (131.52) | 2.94 (2.41) | 1.56 (1.24) | <0.001 | <0.001 |
| Oseltamivir | 98 | 2.97 (2.43-3.63) | 2.95 (2.42-3.60) | 2.95 (125.22) | 2.93 (2.39) | 1.55 (1.23) | <0.001 | <0.001 |
| Amfetamine | 83 | 5.31 (4.27-6.60) | 5.23 (4.22-6.48) | 5.23 (281.76) | 5.18 (4.17) | 2.37 (1.98) | <0.001 | <0.001 |
| Dexamfetamine | 81 | 5.44 (4.36-6.79) | 5.35 (4.31-6.65) | 5.35 (284.92) | 5.31 (4.26) | 2.41 (2.01) | <0.001 | <0.001 |
| Cetirizine | 79 | 2.66 (2.13-3.33) | 2.65 (2.12-3.30) | 2.65 (80.45) | 2.63 (2.11) | 1.40 (1.04) | <0.001 | <0.001 |
| Perampanel | 65 | 17.41 (13.53-22.39) | 16.42 (12.96-20.81) | 16.42 (937.25) | 16.30 (12.67) | 4.03 (3.36) | <0.001 | <0.001 |
| Tezacaftor | 64 | 3.05 (2.38-3.91) | 3.03 (2.37-3.87) | 3.03 (86.62) | 3.01 (2.35) | 1.59 (1.19) | <0.001 | <0.001 |
| Elexacaftor | 61 | 3.81 (2.96-4.91) | 3.77 (2.94-4.85) | 3.77 (123.84) | 3.75 (2.91) | 1.91 (1.47) | <0.001 | <0.001 |
| Dexmethylphenidate | 50 | 6.02 (4.55-7.98) | 5.91 (4.49-7.79) | 5.91 (203.65) | 5.88 (4.44) | 2.56 (2.01) | <0.001 | <0.001 |
| Clonidine | 44 | 2.73 (2.03-3.68) | 2.72 (2.02-3.65) | 2.72 (47.61) | 2.71 (2.01) | 1.44 (0.95) | <0.001 | <0.001 |
| Zanamivir | 35 | 4.10 (2.93-5.72) | 4.05 (2.91-5.63) | 4.05 (80.33) | 4.04 (2.89) | 2.01 (1.41) | <0.001 | <0.001 |
| Desloratadine | 33 | 8.12 (5.74-11.48) | 7.91 (5.65-11.08) | 7.91 (199.14) | 7.88 (5.57) | 2.98 (2.21) | <0.001 | <0.001 |
| Lurasidone | 28 | 5.05 (3.47-7.35) | 4.98 (3.45-7.19) | 4.98 (89.02) | 4.96 (3.41) | 2.31 (1.58) | <0.001 | <0.001 |
| Ziprasidone | 27 | 4.31 (2.95-6.31) | 4.26 (2.93-6.20) | 4.26 (67.40) | 4.25 (2.90) | 2.09 (1.38) | <0.001 | <0.001 |
| Brivaracetam | 20 | 7.36 (4.72-11.48) | 7.19 (4.66-11.09) | 7.19 (106.72) | 7.18 (4.60) | 2.84 (1.83) | <0.001 | <0.001 |
| Testosterone | 18 | 5.65 (3.54-9.01) | 5.55 (3.51-8.78) | 5.55 (67.32) | 5.54 (3.47) | 2.47 (1.49) | <0.001 | <0.001 |
| Beclometasone | 16 | 3.55 (2.17-5.81) | 3.52 (2.16-5.72) | 3.52 (28.85) | 3.51 (2.14) | 1.81 (0.91) | <0.001 | <0.001 |
| Viloxazine | 14 | 5.49 (3.23-9.32) | 5.40 (3.21-9.07) | 5.40 (50.29) | 5.39 (3.18) | 2.43 (1.31) | <0.001 | <0.001 |
| Oxybutynin | 12 | 3.88 (2.19-6.85) | 3.83 (2.19-6.73) | 3.83 (25.20) | 3.83 (2.17) | 1.94 (0.84) | <0.001 | <0.001 |
| Cyproheptadine | 7 | 7.57 (3.57-16.05) | 7.39 (3.56-15.36) | 7.39 (38.81) | 7.39 (3.48) | 2.89 (1.00) | <0.001 | <0.001 |
| Ebastine | 5 | 23.40 (9.38-58.36) | 21.62 (9.32-50.15) | 21.62 (98.65) | 21.61 (8.67) | 4.43 (1.05) | <0.001 | <0.001 |

**Note:** The *p*-value is derived from the chi-squared test in the PRR algorithm. The false discovery rate is the estimated proportion of false positives among the detected signals.

**Abbreviation:** BCPNN, Bayesian confidence propagation neural network; PRR, proportional reporting ratio; ROR, reporting odds ratio; CI, confidence interval; EBGM, empirical Bayes geometric mean; IC, information component; EBGM05, lower limit of the 95% confidence interval for the EBGM; IC025, lower end of the 95% credibility interval for the IC; χ², chi-squared; FDR, false discovery rate.

**Supplementary Table 4. Sensitivity analysis of disproportionality signals for ebastine: Global *vs.* Non-U.S. reports.**

|  | **Global Analysis (Full Database)** | **Non-U.S. Analysis (Sensitivity)** |
| --- | --- | --- |
| a | 5 | 5 |
| b | 58 | 58 |
| c | 7956 | 3457 |
| d | 2159731 | 1011667 |
| ROR | 23.40 | 25.23 |
| RORL (95% CI Lower) | 9.38 | 10.11 |
| RORU (95% CI Upper) | 58.36 | 62.93 |
| PRR | 21.62 | 23.31 |
| χ² (Chi-square) | 98.65 | 106.95 |
| PRRL (95% CI Lower) | 9.32 | 10.04 |
| PRRU (95% CI Upper) | 50.15 | 54.07 |
| EBGM | 21.61 | 23.31 |
| EBGM05 | 8.67 | 10.85 |
| IC | 4.43 | 4.54 |
| IC025 | 1.05 | 0.99 |

**Abbreviation:** ROR, reporting odds ratio; PRR, proportional reporting ratio; BCPNN, Bayesian confidence propagation neural network; EBGM, empirical Bayes geometric mean; 95% CI, 95% confidence interval; χ², chi-squared; IC, information component; IC025, lower end of the 95% credibility interval for the IC; EBGM05, lower limit of the 95% confidence interval for the Empirical Bayes Geometric Mean.
